# ﻿More than 80 years without new taxa: analysis of morphological variation among members of Mexican *Aeneolamia* Fennah (Hemiptera, Cercopidae) support a new species in the genus

**DOI:** 10.3897/zookeys.1139.85270

**Published:** 2023-01-09

**Authors:** Francisco Armendáriz-Toledano, Misael Adrián López-Posadas, Youssef Utrera-Vélez, Jesús Romero Nápoles, Ulises Castro-Valderrama

**Affiliations:** 1 Colección Nacional de Insectos, Departamento de Zoología, Instituto de Biología, Universidad Nacional Autónoma de México, Cto. Zona Deportiva S/N, Ciudad Universitaria, Mexico City, CDMX 04510 Mexico Universidad Nacional Autónoma de México Mexico City Mexico; 2 Universidad de Sonora, Departamento de Agricultura y Ganadería, Km 21 Carretera Hermosillo-Bahía Kino, C.P. 83000, Sonora, Mexico Universidad de Sonora Bahía Kino Mexico; 3 Tecnológico de México-Campus Úrsulo Galván, Km 4.5 Carretera Cardel Chachalacas, Úrsulo Galván, Veracruz, Mexico Tecnológico de México-Campus Úrsulo Galván Úrsulo Galván Mexico; 4 Colegio de Postgraduados, Postgrado en Fitosanidad-Entomología y Acarología, Km. 36.5 México-Texcoco, Montecillo, CP 56230, México State, Mexico Colegio de Postgraduados, Postgrado en Fitosanidad-Entomología y Acarología Texcoco Mexico

**Keywords:** *Aeneolamia* aff. *albofasciata*, Cercopoidea, grasses pest, Spittlebug, sugarcane

## Abstract

The genus *Aeneolamia* includes eight described species and 32 subspecies widely distributed in America. In Mexico, two species (*A.contigua* and *A.albofasciata*) and one subspecies (*A.contiguacampecheana*) are recognized. In a recent study of Cercopidae in Mexico, a new species of *Aeneolamia* was noted from Oaxaca, Mexico based on body color and the ornamentation patterns of tegmen, without a formal taxonomic description. To test the hypothesis of an extant new taxon within the genus a comprehensive analysis of intraspecific morphological variation from 46 morphological features was performed, four related to tegmen color patterns in both sexes, six to male genitalia, and 36 continuous characters measured in specimens of both sexes of Mexican *Aeneolamia* from several geographical localities using traditional univariate, multivariate morphometric, and geometric morphometric methods. This is the first time that this approach has been used in Cercopidae. *Aeneolamiadanpecki* Castro, Armendáriz & Utrera, **sp. nov.** from Oaxaca showed pronounced morphological differences in tegmen coloration patterns, the shape of different elements of the male genitalia, and body measurements compared to the other Mexican members of *Aeneolamia*; therefore, it is described as a new species.

## ﻿Introduction

In the Neotropical region, 60 genera of Cercopidae are integrated into the subfamily Ischnorhininae (Carvalho and Webb 2005; Paladini et al. 2015; Armendáriz-Toledano et al. 2022). One of the most important taxa in this subfamily, due to its economic impact as pests in sugarcane and pastures, is the genus *Aeneolamia* Fennah, 1949, whose members promote considerable losses in crop yields in the countries they inhabit (Urich 1913; Williams 1921; Guagliumi 1962; Oomen 1975; De la Cruz-Llanas et al. 2005; Gómez 2007). The adults of *Aeneolamia* spp. suck the sap of the sugar cane, *Saccharumofficinarum* L., promoting a decrease in the percentage of sucrose compared to canes not damaged by the insects, which causes the sugar extraction process to be less efficient (Williams 1921). Compared with other sugar cane pests in Cercopidae, species of the genus *Aeneolamia* have a shorter development time (Oomen 1975; Peck et al. 2002a; Rodríguez et al. 2002; Sendoya Corrales et al. 2011), which allows a greater number of generations per year and, therefore, increased potential to cause losses (Hernández et al. 2021a, b). For these reasons, *Aeneolamia* spp. have been extensively studied regarding biology (Urich 1913; Williams 1921; Guagliumi 1962; Fewkes1969a, b; Morales 1993; López et al. 2001; Peck et al. 2002a; Sendoya Corrales et al. 2011), taxonomy (Fennah 1949, 1953, 1968; Clark et al. 1976; Carvalho and Webb 2005; Thompson and León González 2005), population dynamics (Urich 1913; Oomen 1975; Jiménez 1978; Wiedijk 1982; Martin et al. 1995; Peck et al. 2002b), distribution (Urich 1913; Guagliumi 1962; Figueredo et al. 2021; Hernández et al. 2021a, b), natural enemies (Urich 1913; Medina et al. 1993; García et al. 2012; Matabanchoy Solarte et al. 2012; Moreno Salguero et al. 2012; Rosero-Guerrero et al. 2012; Obando et al. 2013; Hernández-Domínguez et al. 2016; Grifaldo-Alcántara et al. 2019), economic impact (Williams 1921; Guagliumi 1962; García-García et al. 2006), host plant resistance (Miles et al. 1995; Cardona et al. 2004, 2010; Sotelo-Cardona et al. 2008; Cuarán et al. 2012; Aguirre et al. 2013), and control strategies (Jiménez 1978; Martin et al. 1999; García-García et al. 2006).

Fennah (1949) created the genus *Aeneolamia* with six species, *A.variasemifascia* (Walker, 1851) as the type species and *A.varia* (Fabricius, 1787), *A.colon* (Germar, 1821), *A.contigua* (Walker, 1851), *A.flavilatera* (Urich, 1914), *A.lepidior* (Fowler, 1897), and *A.reducta* (Lallemand, 1924), defined by at least eight morphological features:

1. head with eyes two-thirds as wide as pronotum at widest part, anterolateral margins more or less straight, converging at 110°, width of head between eyes greater than length in middle line (approximately 1.2: 1);
2. fronto-vertex with two deeply impressed lines; ocelli nearer to one another than to eyes, situated on a common prominence;
3. antennae with second segment twice as long as broad, third segment sub globular, or broadly ovoid, both arista placed at same level, shorter arista scarcely longer than third segment;
4. postclypeus moderately inflated, not laterally compressed, distinctly wider across middle in anterior view than at base, in profile shallowly rounded to apex of well-developed median carina, then straight to anteclypeal suture, point of curvature subangulate (approx. 125°), smooth, shining, with setae along grooves;
5. rostrum moderately short, apical joint in anterior view 2.5 × as long as broad;
6. tegmina 2.5 × as long as broad, apical margin broadly rounded, Sc + R forked about level with the union of claval veins, M and Cu united for a short distance near the base, apical venation prominent above general surface, the distal area with very approximately 25 cells, dorsal surface of tegmen often markedly pubescent (2.5 × as long as broad, with apical reticulation);
7. shape of subgenital plates (never greatly elongated, relatively broad, distally transverse, obliquely truncate, or with apicomesal angle produced in a spine);
8. structure of aedeagus (tubular, with one pair of slender strongly deflexed spines attached anteriorly near middle).

In the compilation of Carvalho and Webb (2005), the six species considered by Fennah were reported together with two additional species, *A.albofasciata* (Lallemand, 1939) and *A.sanguiniplaga* (Lallemand, 1938), and more than 30 subspecies. Years later, a new species from Brazil, *A.bucca* Paladini & Cavichioli, 2013 was proposed within the genus (Paladini and Cavichioli 2013). However, it was later assigned by the same authors to the genus *Gervasiella* Paladini & Cavichioli, 2015, based on a cladistic analysis of morphological characters (Paladini and Cavichioli 2015). Currently, *Aeneolamia* includes eight species and 32 described subspecies widely distributed in Brazil, Colombia, Costa Rica, Guatemala, Guyana, Honduras, Mexico, Panama, Venezuela, and Trinidad and Tobago (Carvalho and Webb 2005; Armendáriz-Toledano et al. 2022). In a cladistics framework based on morphological characters, *Aeneolamia* is supported as a sister clade of *Isozulia* Fennah, 1953, and together as the sister group of *Prosapia* Fennah, 1949, within the tribe Tomaspidini (Paladini et al. 2015). The most recent molecular phylogenetic analysis of Ischnorhininae supports *Aeneolamia* and *Isozulia* as sister genera; however, its position within Tomaspidini was separated from *Prosapia* and associated with *Ferorhinella* Carvalho & Webb, 2004, *Aracamunia* Fennah, 1968, and *Tropidorhinella* Schmidt, 1910 (Paladini et al. 2018). Since the description of *A.albofasciata* Lallemand, 1939; *A.flavilaterabelenensis* Guagliumi, 1956; and *A.flavilateraguarici* Guagliumi, 1956 no new species or subspecies have been added to the genus *Aeneolamia*.

In Mexico, *Aeneolamia* is represented by two species, *A.albofasciata* (Lallemand, 1939) and *A.contigua* (Walker, 1851). Both Mexican species of *Aeneolamia* are polyphagous on Poaceae and inhabit almost most regions from Mexican Republic (Martin et al. 1995; López-Collado and Pérez-Aguilar 2012), where they are reported as important damaging pests in sugar cane areas (De la Cruz-Llanas et al. 2005; López-Collado and Pérez-Aguilar 2012; Morales-Pérez et al. 2014; García-González et al. 2017) and grasses (Oomen 1975; Martin et al. 1995; De la Cruz-Zapata et al. 2016). In *A.contigua*, three subspecies have been recognized, from southwestern Mexico: *A.contiguacampecheana* Fennah, 1951 from Haltunchen, Campeche; *A.contiguapostica* (Walker, 1858) from around Orizaba volcano, Veracruz; and *A.contiguasanctaerosae* (Fennah 1953) from Santa Rosa, Yucatan. These subspecies were proposed based on differences in coloration patterns of body and tegmina, without conspicuous differences in male genitalia morphology. In Arméndariz-Toledano et al. (2022), the type specimens of *A.contigua*, *A.contiguapostica*, and *A.contiguasanctaerosae* were compared, leading to the conclusion that these subspecies corresponded only to variations of *A.contigua* in agreement with Clark et al. (1976). In a recent study of the taxonomy and diversity of Cercopidae in Mexico and based on body color and the ornamentation patterns of the tegmina, a new species of *Aeneolamia* was observed from the mountains and central valleys of Oaxaca State. This undescribed taxon was provisionally named Aeneolamiaaff.albofasciata (handwritten label: “Aeneolamiaaff.albofasciata nueva especie”, UCV, deposited in CEAM) for its morphological similarities to *A.albofasciata* (Armendáriz-Toledano et al. 2022). Members of *Aeneolamia* display intra- and interspecific variation in tegmina color, both within and among localities, placing great importance on male genitalia characters as reliable species identifiers, because they are conservative within the species (Fennah 1949; Paladini and Cavichioli 2013). Thus, we tested the hypothesis that A.aff.albofasciata is a new taxon within the genus by analysis of the morphological variation of Mexican *Aeneolamia* species using traditional univariate and multivariate morphometrics of 46 discrete and continuous features of external morphology, tegmina color pattern, and male genitalia on 628 specimens from 59 localities representative of their entire distribution. In addition, we looked for new discrete characters, as well as assessed their usefulness in the identification of these taxa. Furthermore, we performed a geometric morphometric analysis to evaluate whether the variation in the shape of the aedeagus spine allows delimitation of these taxa. This is the first time that this approach has been used to support and define the taxonomic status of a new taxon of Cercopidae. Based on our results, we describe *A.danpecki* sp. nov. and provide a complete dichotomous key to the Mexican species of *Aeneolamia*, replacing the partial key of Armendáriz-Toledano et al. (2022).

## ﻿Materials and methods

A total of 628 *Aeneolamia* adults from 59 Mexican localities corresponding to 260 females and 368 males were reviewed. From the total sample, 64 specimens (43 ♀, 21 ♂) correspond to *A.danpecki* sp. nov., 496 to *A.albofasciata* (178 ♀, 318 ♂), and 68 to *A.contigua* (39 ♀, 29 ♂). For the third species, we included specimens collected around the respective type localities of the previously recognized subspecies *A.contiguacampecheana*, *A.contiguapostica*, and *A.contiguasanctaerosae* because the type localities were not geographically detailed in the original descriptions or the habitat of the subspecies in the locality had disappeared (Table 1). The specimens reviewed were loaned by the following institutions:

**Table 1.** Species, locality, date, and sample size of Mexican *Aeneolamia*. The number of specimens in parentheses refer to those included in the morphometric analysis. *^a^A.albofasciata* identified by Clark in 1975 and deposited in CEAM, *^b^A.contiguacampecheana*, *^c^A.contiguapostica*.

Species	Locality	Date	Total	Female	Male
*A.albofasciata*	Campeche, Colegio de Postgraduados	3/X/2016	199	79 (2)	120
	Campeche, Haltunchen, Km 159.5	2/X/2016	110	10 (1)	100 (2)
	Chiapas, Comunidad Providencia	6/VI/2011	1	–	1 (1)
	Chiapas, ECOSUR, Tapachula	18/VI/1999	1	1	–
	Chiapas, Ejido Rizo de Oro, Cintalapa	27/V/2011	2	–	2
	Guerrero, Acapulco	21/VIII/1938	1	1	–
	Guerrero, Petaquillas, 9 km W Chilpancingo	6/VI/1963	1	–	1
	Michoacán, Charapendo	18/VIII/2015	1	1 (1)	–
	Michoacán, Tangamandapio	14/IX/2017	119	56 (3)	63 (5)
	Michoacán, Taretan	13/IX/1963	2	2 (1)	–
	Michoacán, Uruapán	VII/1998	1	–	1 (1)
	Morelos, Cuatla	14/IX/1980	3	1 (1)	2
	Morelos, Cuautla, Cuautlixco	22/V/2002	1	–	1 (1)
	Morelos, Palo Bolero	7/X/1995	1	–	1 (1)
	Nuevo León, Apodaca	4/VIII/1979	20	6	14
	Quintana Roo, Tecnológico de Chetumal	15/X/2017	2	–	2
	San Luis Potosí, Sierra El Abra, Los Patos	8/IX/2017	1	1	–
	Sonora, Municipio de ímuris, ímuris	9/VIII/2013	1	–	1
	Sonora, Centro Invest. Pec. Est. Sonora, Carbó	X/1981	2	2	–
	Tabasco, Cárdenas	28/VI/1982	1	1 (1)	–
	Tabasco, Cárdenas	12/I/2012	1	1 (1)	–
	Tamaulipas, Cd. Mante	VI/1987	1	1 (1)	–
	Tamaulipas, Cd. Mante	7/X/1983	4	1 (1)	3 (1)
*^a^*	Veracruz, Cosamaloapan	20/VII/1962	1	1	–
*^a^*	Veracruz, Tecolutla	7/IX/1973	1	–	1
	Veracruz, Km 4.5 Carr Cardel-Salmoral	4/IX/2003	14	12 (1)	2
	Veracruz, Úrsulo Galván	23/VI/2021	4	1 (1)	3 (3)
	Total		496	178 (15)	318 (15)
*A.contigua*	Quintana Roo, 2 km S Rancho El 24	20/XII/1984	1	–	1
	Chiapas, Calzada larga, Villaflores	27/X/2012	1	–	1
	Chiapas, Finca Cucalhuitz, 19 Km NE Bochil	28/XI/1961	1	1 (1)	–
	Chiapas, Llano La Lima, Tapachula	9/VI/2013	1	1	–
	Chiapas, Palenque	31/I/1985	1	1	
	Chihuahua, Chihuahua	12/VII/1938	1	–	1
	Guerrero, Almolonga	30/VII/1962	5	1 (1)	4 (3)
	Jalisco, Ameca	23/VII/1999	1	–	1
	Michoacán, Morelia	1/VI/1963	1	–	1 (1)
	Morelos, Cuatla	6/II/1996	2	1 (1)	1 (1)
	Morelos, Reserva de la Biosfera Huahutla	27/V/2000	15	13 (3)	2
	Nayarit, Guayabitos	2/X/1980	1	1 (1)	–
	Oaxaca, I. Bastida	12/IX/1981	1	–	1 (1)
	Oaxaca, Tehuantepec	10/VII/1966	1	1 (1)	–
*^b^*	Quintana Roo, Tecnológico de Chetumal	15/X/2017	1	–	1
	Tabasco, Cárdenas	14/VII/1994	1	–	1 (1)
*^c^*	Veracruz, Cd. Mendoza	15/IX/1994	2	1	1 (1)
*^c^*	Veracruz, Colegio de Postgraduados, Campus Córdoba	15/IX/1994	1	–	1 (1)
	Veracruz, Est. Los Tuxtlas, San Andrés	21/IX/2007	1	–	1 (1)
	Veracruz, Isla	13/VII/2002	2	2 (2)	–
	Veracruz, Km 14 Aut. Cárdenas-Minatitlán, Rancho La Majada	4/X/2016	15	10 (2)	5 (4)
*^c^*	Veracruz, La Antigua	28/VIII/1978	2	2 (2)	–
	Veracruz, Las Vigas	19/VI/1965	2	1	1 (1)
	Veracruz, Playa Escondida, Catemaco	13/VI/1979	1	1 (1)	–
	Veracruz, Playa Escondida, Catemaco	13/VI/1979	1	–	1
	Veracruz, Tinája	18/IX/1994	1	–	1
	Yucatán, Chichen Itza	20/VI/1985	2	1	1
	Yucatán, Ruta 295, Km 93 Rio Lagartos	18/VI/1985	2	–	2
*^b^*	Yucatán, Cuncunul	13/IX/1994	1	1	–
	Total		68	39 (15)	29 (15)
*Aeneolamiadanpecki* sp. nov.	Oaxaca, 5 km San Martín Lochila	12/VII/2004	1	1 (1)	–
	Oaxaca, Sola de Vega	28/IX/2003	48	33 (5)	15 (9)
	Oaxaca, La Trinidad, Zaachila	28/VIII/2018	15	9 (9)	6 (6)
	Total		64	43 (15)	21 (15)
TOTAL			**628**	**260**	**368**

**CEAM**Colección de Insectos del Colegio de Postgraduados, Montecillo, Texcoco, México;

**CNIN**Colección Nacional de Insectos del Instituto de Biología de la Universidad Nacional Autónoma de México, México City, Mexico.

Taxonomic identifications of the species were based on male genitalia. In addition, we included two specimens identified as *A.albofasciata* (= *A.albofasciataoccidentalis*) from CEAM (Table 1) and determined by W. E. Clark in 1975, an authority on the identification of *Aeneolamia* species. *Aeneolamiadanpecki* sp. nov. was recognized by a dark brown to light brown tegmen, with two incomplete and barely visible transverse bands, one of them oblique on the basal third and another straight on the distal third, or only the basal band visible, or both absent. Males and females were recognized by their genitalia.

To manipulate specimens and take photographs, we used the method of Valdez-Carrasco (Castro-Valderrama et al. 2018). The pygofer of some males of each taxon was detached and clarified in 10% KOH solution for 12–24 h; after which the KOH was neutralized with acetic acid and washed with distilled water. Photographs of the genitalia were taken with a Leica MZ8 stereomicroscope, connected with Nikon E5700 camera and E5700 v. 1.1 software to capture images. Photographs of adult habitus were taken with a digital Olympus E-620 camera attached to an Olympus SZX7 stereoscope and images were captured with Olympus Studio 2.22 software. Images were stacked with COMBINE ZP free software and edited with GIMP 2.8.14 free software. Morphological terminology follows and is adapted from Fennah (1949, 1953, 1968), Nast (1950), Hamilton (1977), Paladini and Cavichioli (2015), and Le Cesne et al. (2021).

**Figure 1.**
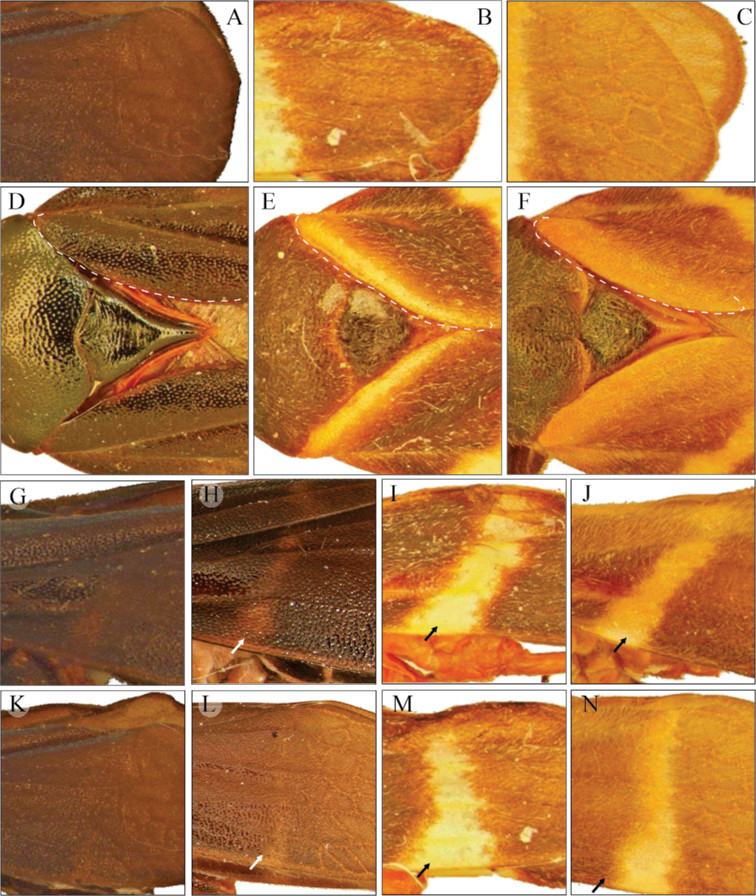
Character states to compare the variation of tegmen color patterns **A, D, G, H, K, L***Aeneolamiadanpecki* sp. nov. **B, E, I, M***A.albofasciata***C, F, J, N***A.contigua***D–F** dotted lines indicate the internal anterior region of tegmen. Arrows indicate the transversal lines on tegmen: **G–J** anterior region **K–N** posterior region.

**Figure 2.**
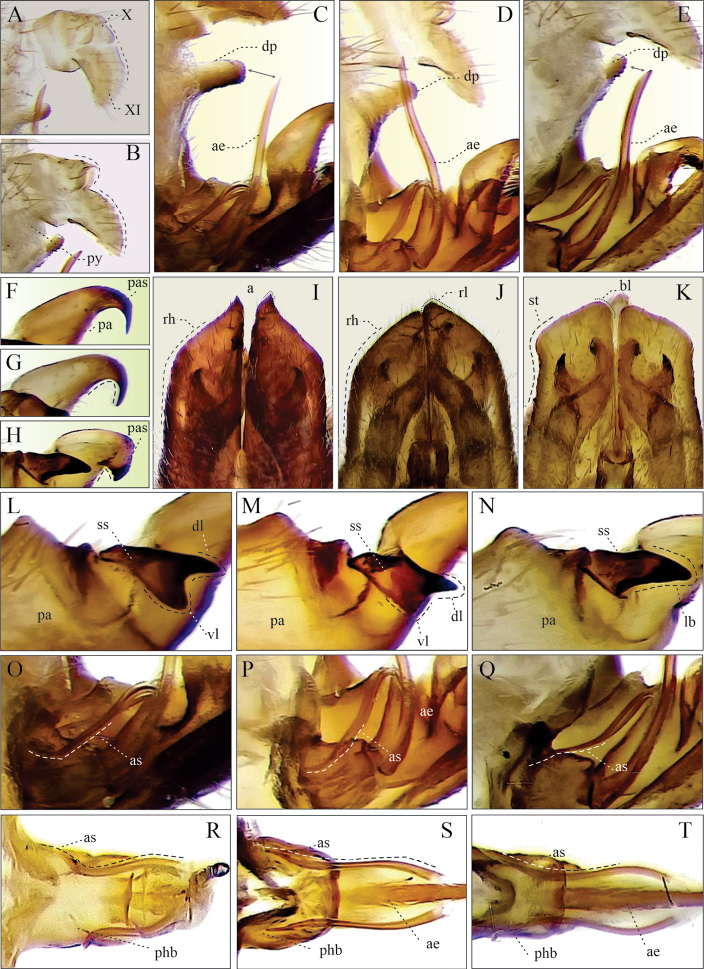
Character states to compare the variation of male genitalia **A, C, F, I, L, O, R***Aeneolamiadanpecki* sp. nov. **D, G, J, M, P, S***A.albofasciata***B, E, H, K, N, Q, T***A.contigua***A, B** anal tube, lateral view **C–E** aedeagus within pygofer, lateral view **F–H** distal region of parameres, lateral view **I–K** subgenital plates, ventral view **L–N** subapical spines of parameres, lateral view **O–Q** phallobase and aedeagus, lateral view **R–T** phallobase and aedeagus, anterodorsal view. Abbreviations: **ae** aedeagus, **a** acuminate, **as** aedeagus spine, **bl** blunt internal distal edge, **dl** dorsal lobe, **dp** digital process of pygofer, **lb** lobe, **pa** paramere, **pas** primary apical spine of parameres, **phb** phallobase, **py** pygofer, **rh** rhomboidal apex with lateral straight edge, **ss** secondary subapical spine of paramere, **st** slight concave lateral edge, **vl** ventral inferior lobe, **X** tenth segment of anal tube, **XI** eleventh segment of anal tube.

**Figure 3.**
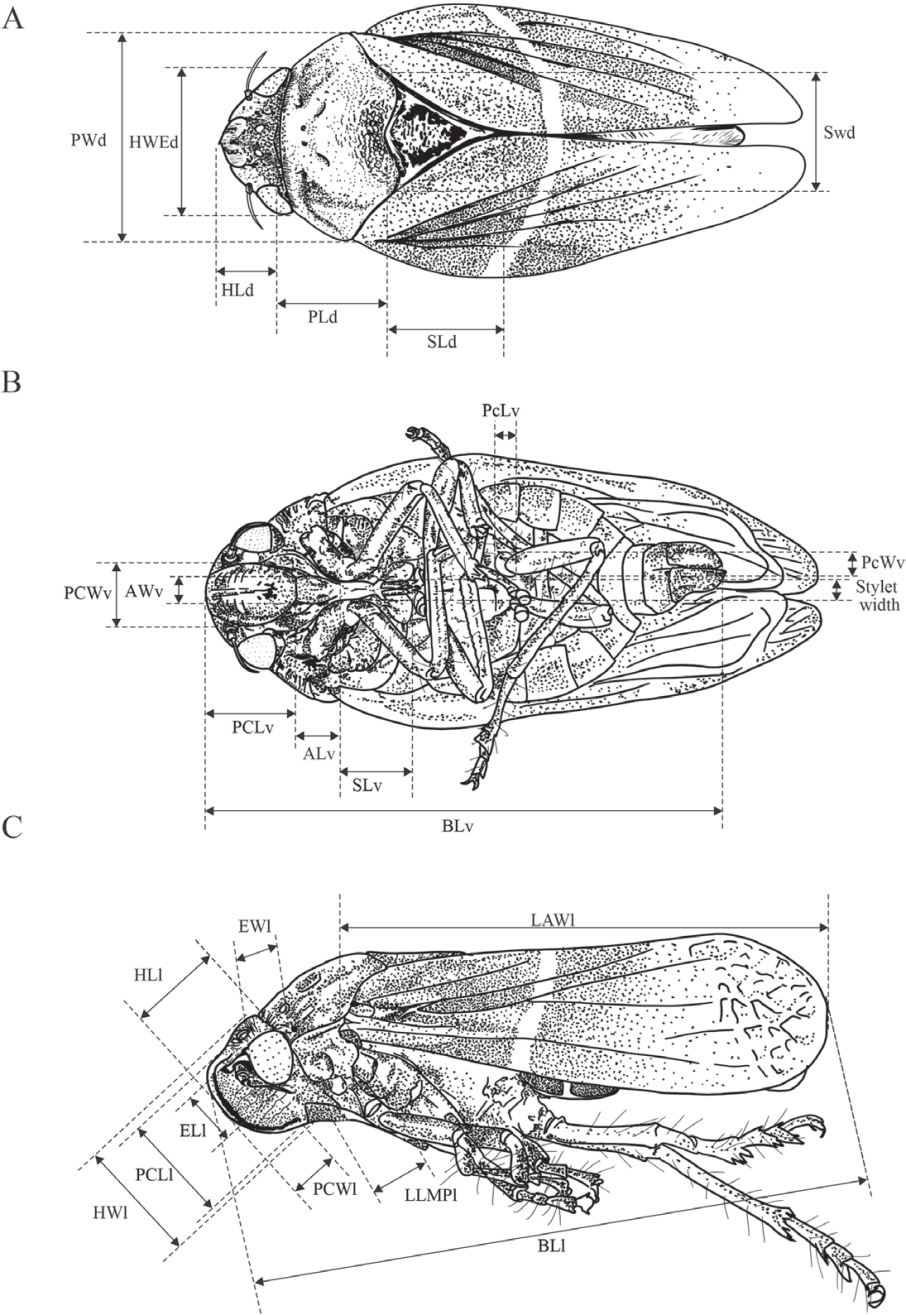
Distribution and details of the continuous features used to quantify the morphological variation of Mexican *Aeneolamia* spp. Habitus view: **A** dorsal **B** ventral **C** lateral. Features Abbreviations: **HWE_d_** Head width with eyes **HL_d_** head length in dorsal view, **PW_d_** pronotum width in dorsal view **PL_d_** pronotum length in dorsal view **SW_d_** scutellum width in dorsal view **SL_d_** scutellum length in dorsal view **PcL_v_** postclypeus length in ventral view **PcW_v_** postclypeus width in ventral view **Al_v_** anteclypeus length in ventral view **AW_v_** anteclypeus width in ventral view **SL_v_** stylet length in ventral view **SW_v_** stylet width in ventral view **PCW_v_** posterior coxa width in ventral view **PcL_v_** posterior coxa length in ventral view **BLW_v_** body length without wings in ventral view **PCL_l_** postclypeus length in lateral view **PCW_l_** postclypeus width in lateral view **EL_l_** eye length in lateral view **Ew_l_** eye width in lateral view **HL_l_** head length in lateral view **HW_l_** head width in lateral view **LLMP_l_** length of lateral margin pronotum in lateral view **BL_l_** body length including wings in lateral view **LAW_l_** length of the anterior wing in lateral view **WLH_d_** width-length radio of head in dorsal view **WLP_d_** width-length ratio of pronotum in dorsal view **WLS_d_** width-lengh radio of scutellum in dorsal view **WLC_v_** width-length radio of clypeus **PR_v_** postclypeus ratio in ventral view (width/length) **SR_v_** stylet ratio in ventral view (width/length) **RCR_v_** coxa ratio (width/length) **RBW_l_** ratio between body length with wings and length without wings **RPC_l_** postclypeus radio in lateral view (width/length) **Re_l_** eye ratio in lateral view (width/length) **HRAL_l_** head ratio in lateral view (width/length) **BLW_l_** ratio between the length of body with wings and forewing length in lateral view.

**Figure 4.**
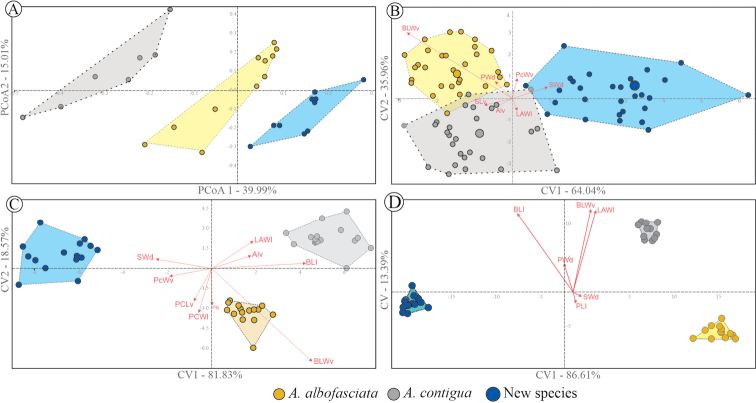
Scatter plots of ordination analyses to evaluate the morphological variation of Mexican *Aeneolamia* spp.: **A** principal coordinate analysis using the 10 discrete (male tegmina color pattern and male genitalia) and 36 continuous features of males **B** analysis of canonical variation with 36 morphological continuous features of both sexes **C** females **D** males. In the center of the scatter plot, vectors corresponding to the contribution of the traits in the multivariate space (**B, C, D**). The largest circles are the centroid of the polygons (B). Abbreviations: **Al_v_** Anteclypeus length in ventral view **BL_s_** body length without wings in ventral view **BLW_v_** body length without wings in ventral view **LAW_l_** length of the anterior wing in lateral view **PCW_v_** postclypeus width in ventral view **PL_d_** postclypeus length in dorsal view **PW_d_** pronotum width in dorsal view **PcW_s_** postclypeus width in lateral view **SW_d_** scutellum width in dorsal view **SL_v_** stylet length in ventral view.

*Discrete morphological characters*. Because of high polymorphism in color patterns of wings recorded in some species of Auchenorrhyncha families, particularly in cercopids, and due to the male genitalia traits providing robust evidence to support *A.danpecki* sp. nov. (Paladini et al. 2015), a comparison of the variations of tegmina color patterns and male genitalia morphology was performed among *Aeneolamia* species. Tegmen color patterns were analyzed in the entire sample (*n* = 628), and male genitalia features from ten specimens of *A.danpecki* sp. nov., eleven specimens of *A.albofasciata*, and seven specimens of *A.contigua*. These characters are as follows:

1. Tegmen color (**TC**). Lateral and dorsal view. (1) Black (Fig. 1A, D), (2) dark brown (Fig. 1B, E), (3) light brown (Fig. 1C).
2. Color of internal clavus edge in the tegmen (**CIE**). Dorsal view. (1) Same color as tegmen (Fig. 1D), (2) a yellowish or white line (Fig. 1E), (3) an orange or red line (Fig. 1F).
3. Color pattern on the anterior third of tegmen (**CAT**). Lateral view. (1) Same color as tegmen (Fig. 1G), (2) an inconspicuous thin transversal line (Fig. 1H), (3) a conspicuous broad transversal yellowish or white line (Fig. 1I), (4) a conspicuous broad transversal orange or red line (Fig. 1J).
4. Color pattern on the distal third of tegmen (**CDT**). Lateral view. (1) The same color as tegmen (Fig. 1K), (2) an incomplete thin transversal line (Fig. 1L), (3) a complete broad transversal yellowish or white line (Fig. 1M), (4) a complete broad transversal orange or red line (Fig. 1N).
5. Elevation of the anal tube sclerites (**EAE**). (1) tenth and eleventh tergites at the same level on the horizontal (Fig. 2A), (2) tenth tergite higher than the eleventh, with respect to the horizontal (Fig. 2B).
6. Shape of subgenital plates (**SGP**). Ventral view. (1) Acute apex with straight lateral edges and rhomboidal to the apex (Fig. 2I, J), (2) apex obliquely truncate with lateral edges slightly concave (Fig. 2K).
7. Shape of internal edge of subgenital plate apex (**SEGP**). Ventral view. (1) Acuminate in a pointed lobe (Fig. 2I), (2) acuminate in a rounded lobe (Fig. 2J), (3) blunt (Fig. 2K).
8. Paramere, shape of primary apical spine (**ASP**). Lateral view. (1) Long and thin spine with a continuous curvature not angulated (Fig. 2F, G), and (2) short and wide spine with a pronounced angulated curvature (Fig. 2H).
9. Parameter, shape of secondary subapical spine (**SSP**). Lateral view. (1) Two rounded acute lobes similar in size and shape (Fig. 2L), (2) two acute lobes, the dorsal one conspicuously bigger than the ventral (Fig. 2M), (3) one big lobe (Fig. 2N).
10. Tip of aedeagus spines (**PRE**). (1) Lateral view, conspicuously curved upward and touching the superior margin of phallobase, bent to form an almost 90° angle (Figs 2O, 8A, B), and dorsal view, conspicuously sinuous (Fig. 2R); (2) lateral view, slightly curved upward (Figs 2P, 8C) and dorsal view, slightly sinuous (Fig. 2S); (3) lateral view, slightly curved downward (Figs 2Q, 8D) and dorsal view, conspicuously sinuous (Fig. 2T).

### ﻿Continuous quantitative morphological characters

Because the *Aeneolamia* species display apparent differences in body size, 90 adults from 31 Mexican localities were compared using measurements of the head, mouthparts, pronotum, tegmina, and legs (Fig. 3). Using these features, a comparison of the morphological variation among *A.danpecki* sp. nov. (*n* = 15♂, 15♀), *A.albofasciata* (*n* = 15♂, 15♀), and *A.contigua* (*n* = 15♂, 15♀) could be performed. These characters are as follows:

11) Head width with eyes (**HWE_d_**) , 12) head length in dorsal view (**HL_d_**), 13) pronotum width in dorsal view (**PW_d_**), 14) pronotum length in dorsal view (**PL_d_**), 15) scutellum width in dorsal view (**SW_d_**), 16) scutellum length in dorsal view (**SL_d_**), 17) postclypeus length in ventral view (**PcL_v_**), 18) postclypeus width in ventral view (**PcW_v_**), 19) anteclypeus length in ventral view (**Al_v_**), 20) anteclypeus width in ventral view (**AW_v_**), 21) stylet length in ventral view (**SL_v_**), 22) stylet width in ventral view (**SW_v_**), 23) posterior coxa width in ventral view (**PCW_v_**), 24) posterior coxa length in ventral view (**PcL_v_**), 25) body length without wings in ventral view (**BLW_v_**), 26) postclypeus length in lateral view (**PCL_l_**), 27) postclypeus width in lateral view (**PCW_l_**), 28) eye length in lateral view (**EL_l_**), 29) eye width in lateral view (**Ew_l_**), 30) head length in lateral view (**HL_l_**), 31) head width in lateral view (**HW_l_**), 32) length of lateral margin pronotum in lateral view (**LLMP_l_**), 33) body length including wings in lateral view (**BL_l_**), 34) length of the anterior wing in lateral view (**LAW_l_**), 35) width-length radio of head in dorsal view (**WLH_d_**), 36) width-length ratio of pronotum in dorsal view (**WLP_d_**), 37) width-lengh radio of scutellum in dorsal view (**WLS_d_**), 38) width-length radio of clypeus (**WLC_v_**), 39) postclypeus ratio in ventral view (width/length) (**PR_v_**), 40) stylet ratio in ventral view (width/length) (**SR_v_**), 41) coxa ratio (width/length) (**RCR_v_**), 42) ratio between body length with wings and length without wings (**RBW_l_**), 43) postclypeus radio in lateral view (width/length) (**RPC_l_**), 44) eye ratio in lateral view (width/length) (**RE_l_**), 45) head ratio in lateral view (width/length) (**HRAL_l_**), 46) and ratio between the length of body with wings and forewing length in lateral view (**BLW_l_**).

### ﻿Data analyses

The frequency of character states for each feature was calculated for each taxon in the contingency tables (Tables 2, 3). To evaluate if the differences in frequency among character states are associated with different taxa, both Chi-square Test and the contingency coefficient were performed (e.g., Zar 2010). The normality of the distribution for the quantitative continuous features was independently tested by Shapiro and Wilkinson’s test; these features were log-transformed to meet the criteria of normality. Basic descriptive statistics were calculated (mean and standard deviation) and the variation of each character was compared among species and between sexes. To determine whether each characteristic differed between sexes and putative species, we performed a two-way analysis of variance (ANOVA) with sex and species as factors, and multiple comparisons with a Tukey test (Zar 2010), but we only provide values that were significantly different at the 5% level (Tables 4, 5, 6).

**Table 2.** Frequencies of multi-state or binary characters used to compare the variation of color patterns of tegmen among Mexican *Aeneolamia* species. Abbreviations: **TC** tegmen color **CIE** color of internal clavus edge in the tegmen **CAT** color pattern on the anterior third of tegmen **CDT** color pattern on the distal third of tegmen.

Attribute	Character states	*A.danpecki*	*A.albofasciata*	*A.contigua*	Chi^2^:	CC**
1.- **TC**	(1) Black.	50 (78%)	362 (73%)	0	180.5*	0.62
	(2) Dark brown.	14 (22%)	134 (27%)	48 (70%)		
	(3) Light brown.	0	0	20 (30%)		
2.- **CIE**	(1) Same color as tegmen.	64 (100%)	397 (80%)	0	300.94*	0.73
	(2) With a yellowish or white line.	0	99 (20%)	0		
	(3) With an orange line.	0	0	68 (100%)		
3.- **CAT**	(1) The same color as tegmen.	54 (85%)	0	0	500*	0.81
	(2) Incomplete thin transversal line.	10 (15%)	0	0		
	(3) Complete broad transversal yellowish or white line.	0	494 (100%)	0		
	(4) Complete broad transversal orange or red line.	0	0	68 (100%)		
4.- **CDT**	(1) The same color as tegmen.	54 (85%)	0	0	600*	0.81
	(2) With an incomplete thin transversal line.	10 (15%)	0	0		
	(3) With a complete broad transversal. yellowish or white line.	0	494 (100%)	0		
	(4) With a complete broad transversal orange or red line.	0	0	68 (100%)		
	*n* =	64	494	68		

** CC contingency coefficient: * p ≤ 0.001 the significance of association of frequency among character states and taxa.

**Table 3.** Frequencies of multi-state or binary characters used to compare the variation male genitalia among Mexican *Aeneolamia* species. Abbreviations: **EAE** elevation of the anal tube sclerites **SGP** shape of subgenital plates **SEGP** shape of internal edge of subgenital plate apex **ASP** paramere, shape of primary apical spine **SSP** parameter, shape of secondary subapical spine **PRE** tip of aedeagus spines.

Attribute	Character state	*A.danpecki*	*A.albofasciata*	*A.contigua*	Chi2:	CC**
5.- **EAE**	(1) Tenth and eleventh tergites at the same level on the horizontal.	0	7 (58%)	0		
	(2) Tenth higher than the eleventh tergite, with respect to the horizontal.	10 (100%)	5 (42%)	7 (100%)	143.8*	0.56
6.- **SGP**	(1) Acute apex with straight lateral edges.	10 (100%)	12(100%)	0		
	(2) Apex obliquely truncate with lateral edges slightly concave.	0	0	7 (100%)		
7.- **SEGP**	(1) Acuminate in a pointed lobe.	10 (100%)	0	0		
	(2) Acuminate in a rounded lobe.	0	12 (100%)	0		
	(3) Blunt.	0	0	7 (100%)	300*	0.7
8.- **ASP**	(1) Long and thin spine with a continuous curvature not angulated.	10 (100%)	12 (100%)	0		
	(2) Short and wide spine with a pronounced angulated curvature.	0	0	7 (100%)	300*	0.7
9.- **SSP**	(1) With two rounded acute lobes similar in size and shape.	10 (100%)	0	0		
	(2) With two acute lobes, the dorsal one conspicuously bigger than the ventral.	0	12 (100%)	0		
	(3) With one big lobe.	0	0	7 (100%)	600*	0.81
10.- **PRE**	(1) Conspicuously curved upward.	10 (100%)	0	0		
	(2) Slightly curved upward.	0	12 (100%)	0		
	(3) Slightly curved downward.	0	0	7 (100%)	414*	0.81
	*n* =	10	11	7		

** CC contingency coefficient: * p ≤ 0.001 the significance of association of frequency among character states and taxa.

**Table 4.** Measurements of morphological characteristics of three Mexican *Aeneolamia* spp. as mean ± standard deviation (mm); * Features that display statistically significant differences among species supported by two way ANOVA; in these cases mean values with the same letter were not significantly different at the 5% level by the Tukey test. Abbreviations: **HWE_d_** Head width with eyes **HL_d_** head length in dorsal view, **PW_d_** pronotum width in dorsal view **PL_d_** pronotum length in dorsal view **SW_d_** scutellum width in dorsal view **SL_d_** scutellum length in dorsal view **PcL_v_** postclypeus length in ventral view **PCW_v_** postclypeus width in ventral view **Al_v_** anteclypeus length in ventral view **AW_v_** anteclypeus width in ventral view **SL_v_** stylet length in ventral view **SW_v_** stylet width in ventral view **PcW_v_** posterior coxa width in ventral view **PcL_v_** posterior coxa length in ventral view **BLW_v_** body length without wings in ventral view **PCL_l_** postclypeus length in lateral view **PCW_l_** postclypeus width in lateral view **EL_l_** eye length in lateral view **Ew_l_** eye width in lateral view **HL_l_** head length in lateral view **HW_l_** head width in lateral view **LLMP_l_** length of lateral margin pronotum in lateral view **BL_l_** body length including wings in lateral view **LAW_l_** length of the anterior wing in lateral view **WLH_d_** width-length radio of head in dorsal view **WLP_d_** width-length ratio of pronotum in dorsal view **WLS_d_** width-lengh radio of scutellum in dorsal view **WLC_v_** width-length radio of clypeus **PR_v_** postclypeus ratio in ventral view (width/length) **SR_v_** stylet ratio in ventral view (width/length) **RCR_v_** coxa ratio (width/length) **RBW_l_** ratio between body length with wings and length without wings **RPC_l_** postclypeus radio in lateral view (width/length) **REl** eye ratio in lateral view (width/length) **HRAL_l_** head ratio in lateral view (width/length) **BLW_l_** ratio between the length of body with wings and forewing length in lateral view.

Attribute Abbreviation (mm)	*A.danpecki*	*A.albofasciata*	*A.contigua*
11.-**HWE_d_**	1.90 ± 0.07	2.04 ± 0.03	1.96 ± 0.07
12.-**HL_d_**	0.87 ± 0.03	0.94 ± 0.01	0.91 ± 0.04
13.-**PW_d_**	2.73 ± 0.10	2.90 ± 0.04	2.81 ± 0.10
14.-**PL_d_**	1.62 ± 0.06	1.75 ± 0.02	1.68 ± 0.06
15.-**SW_d_**	1.24 ± 0.05	1.18 ± 0.01	1.14 ± 0.04
16.-SL_d_*	1.43**^c^** ± 0.05	1.57**^b^** ± 0.02	1.61**^a^** ± 0.02
17.-**PcL_V_**	1.24 ± 0.04	1.30 ± 0.02	1.22 ± 0.04
18.-**PCW_v_**	0.83 ± 0.05	0.83 ± 0.02	0.77 ± 0.03
19.-Al_v_*	0.70**^b^** ± 0.02	0.76**^a^** ± 0.01	0.78**^a^** ± 0.03
20.-**AW_v_**	0.48 ± 0.02	0.53 ± 0.02	0.48 ± 0.02
21.-SL_v_*	0.76**^b^** ± 0.03	0.77**^b^** ± 0.01	0.82**^a^** ± 0.01
22.-SW_v_*	0.19**^b^** ± 0.00	0.22**^a^** ± 0.01	0.21**^a^** ± 0.01
23.-**PcW_v_**	0.49 ± 0.02	0.54 ± 0.01	0.52 ± 0.02
24.-**PcL_v_**	0.59 ± 0.02	0.62 ± 0.01	0.59 ± 0.02
25.-BLW_v_*	6.33**^c^** ± 0.23	7.01**^a^** ± 0.09	6.67**^b^** ± 0.23
26.-**PCL_I_**	0.73 ± 0.03	0.76 ± 0.02	0.71 ± 0.02
27.-**PCW_I_**	1.17 ± 0.04	1.24 ± 0.03	1.18 ± 0.02
28.-**El_I_**	0.47 ± 0.02	0.51 ± 0.01	0.49 ± 0.02
29.-**Ew_I_**	0.63 ± 0.02	0.68 ± 0.01	0.67 ± 0.02
30.-**HL_I_**	0.90 ± 0.03	0.92 ± 0.02	0.87 ± 0.03
31.-**HW_I_**	1.34 ± 0.05	1.40 ± 0.02	1.38 ± 0.02
32.-LLMP_I_*	0.65**^c^** ± 0.03	0.72**^a^** ± 0.02	0.68**^b^** ± 0.02
33.-**BL_I_**	7.91 ± 0.27	8.21 ± 0.12	8.21 ± 0.28
34.-**LAW_I_**	6.42 ± 0.22	6.57 ± 0.08	6.64 ± 0.22
35.-**WLH_d_**	0.14 ± 0.00	0.14 ± 0.00	0.13 ± 0.01
36.-**WLP_d_**	0.10 ± 0.00	0.11 ± 0.00	0.10± 0.00
37.-**WLS_d_**	0.05 ± 0.00	0.05 ± 0.00	0.05 ± 0.00
38.-**WLC_v_**	0.09 ± 0.00	0.10 ± 0.00	0.1 ± 0.00
39.-**PR_v_**	0.09 ± 0.00	0.10 ± 0.00	0.1 ± 0.00
40.-**SR_v_**	0.25 ± 0.01	0.24 ± 0.00	0.25 ± 0.01
41.-**RCR_v_**	0.05 ± 0.00	0.06 ± 0.00	0.05 ± 0.00
42.-**RBW_l_**	0.08 ± 0.00	0.08 ± 0.00	0.07 ± 0.00
43.-**RPC_l_**	0.04 ± 0.00	0.04 ± 0.00	0.03 ± 0.00
44.-**RE_l_**	0.05 ± 0.00	0.05 ± 0.00	0.04 ± 0.00
45.-**HRAL_l_**	0.05 ± 0.00	0.04 ± 0.00	0.04 ± 0.00
46.-**BLW_l_**	0.08 ± 0.00	0.08 ± 0.00	0.07 ± 0.00

**Table 5.** Measurements of morphological characteristics of three Mexican *Aeneolamia* spp. (Mean and standard deviation (mm)); * Features that display significant statistical differences among interaction species-sexes supported by two-way ANOVA, in each species, mean values with the same letter were not significantly different at the 5% level by Tukey test. Abbreviations: **HWE_d_** Head width with eyes **HL_d_** head length in dorsal view, **PW_d_** pronotum width in dorsal view **PL_d_** pronotum length in dorsal view **SW_d_** scutellum width in dorsal view **SL_d_** scutellum length in dorsal view **PcL_v_** postclypeus length in ventral view **PCW_v_** postclypeus width in ventral view **Al_v_** anteclypeus length in ventral view **AW_v_** anteclypeus width in ventral view **SL_v_** stylet length in ventral view **SW_v_** stylet width in ventral view **PcW_v_** posterior coxa width in ventral view **PcL_v_** posterior coxa length in ventral view **BLW_v_** body length without wings in ventral view **PCL_l_** postclypeus length in lateral view **PCW_l_** postclypeus width in lateral view **EL_l_** eye length in lateral view **Ew_l_** eye width in lateral view **HL_l_** head length in lateral view **HW_l_** head width in lateral view **LLMP_l_** length of lateral margin pronotum in lateral view **BL_l_** body length including wings in lateral view **LAW_l_** length of the anterior wing in lateral view **WLH_d_** width-length radio of head in dorsal view **WLP_d_** width-length ratio of pronotum in dorsal view **WLS_d_** width-lengh radio of scutellum in dorsal view **WLC_v_** width-length radio of clypeus **PR_v_** postclypeus ratio in ventral view (width/length) **SR_v_** stylet ratio in ventral view (width/length) **RCR_v_** coxa ratio (width/length) **RBW_l_** ratio between body length with wings and length without wings **RPC_l_** postclypeus radio in lateral view (width/length) **RE_l_** eye ratio in lateral view (width/length) **HRAL_l_** head ratio in lateral view (width/length) **BLW_l_** ratio between the length of body with wings and forewing length in lateral view.

Attribute Acronym (mm)	*A.danpecki*		*A.albofasciata*		*A.contigua*	
	♀	♂	♀	♂	♀	♂
11.-HWE_d_*	2.06**^a^** ± 0.01	1.75**^b^** ± 0.12	2.13 ± 0.028	1.95 ± 0.04	2.11**^a^** ± 0.02	1.81**^b^** ± 0.12
12.-HL_d_*	0.95**^a^** ± 0.01	0.79**^b^** ± 0.05	1.00 ± 0.014	0.89 ± 0.01	1.02**^a^** ± 0.05	0.81**^b^** ± 0.06
13.-PW_d_*	2.95**^a^** ± 0.03	2.50**^b^** ± 0.17	3.01 ± 0.039	2.78 ± 0.05	3.00 ± 0.04	2.62 ± 0.18
14.-**PL_d_**	1.74 ± 0.02	1.49 ± 0.10	1.82 ± 0.028	1.68 ± 0.03	1.80 ± 0.02	1.57 ± 0.11
15.-SW_d_*	1.35**^a^** ± 0.04	1.13**^b^** ± 0.08	1.23 ± 0.015	1.13 ± 0.02	1.20 ± 0.02	1.09 ± 0.08
16.-SL_d_*	1.58**^a^** ± 0.03	1.28**^b^** ± 0.09	1.64 ± 0.031	1.49 ± 0.02	1.61 ± 0.02	1.44 ± 0.11
17.-PcL_V_*	1.35**^a^** ± 0.01	1.13**^b^** ± 0.08	1.37 ± 0.028	1.22 ± 0.02	1.29 ± 0.03	1.15 ± 0.08
18.-PCW_v_*	0.97**^a^** ± 0.06	0.69**^b^** ± 0.05	0.91 ± 0.025	0.75 ± 0.02	0.85**^a^** ± 0.03	0.70**^b^** ± 0.05
19.-Al_v_*	0.74 ± 0.01	0.66 ± 0.05	0.79 ± 0.019	0.73 ± 0.02	0.86**^a^** ± 0.01	0.72**^b^** ± 0.05
20.-AW_v_*	0.54**^a^** ± 0.01	0.42**^b^** ± 0.03	0.58**^a^** ± 0.025	0.47**^b^** ± 0.01	0.55**^a^** ± 0.01	0.43**^b^** ± 0.03
21.-SL_v_*	0.82**^a^** ± 0.02	0.71**^b^** ± 0.05	0.81 ± 0.021	0.73 ± 0.01	0.84 ± 0.02	0.75 ± 0.06
22.-**SW_v_**	0.20 ± 0.00	0.20 ± 0.00	0.23 ± 0.009	0.21 ± 0.00	0.23 ± 0.01	0.19 ± 0.01
23.-PcW_v_*	0.55**^a^** ± 0.01	0.43**^b^** ± 0.03	0.58 ± 0.011	0.50 ± 0.01	0.57**^a^** ± 0.01	0.43**^b^** ± 0.04
24.-PcL_v_*	0.64 ± 0.01	0.55 ± 0.04	0.65 ± 0.013	0.59 ± 0.02	0.64**^a^** ± 0.01	0.50**^b^** ± 0.05
25.-BLW_v_*	6.91**^a^** ± 0.08	5.70**^b^** ± 0.39	7.27 ± 0.115	6.74 ± 0.11	7.15 ± 0.10	6.21 ± 0.43
26.-PCL_I_*	0.79**^a^** ± 0.02	0.68**^b^** ± 0.05	0.85**^a^** ± 0.017	0.67**^b^** ± 0.00	0.78**^a^** ± 0.02	0.66**^b^** ± 0.02
27.-PCW_I_*	1.27**^a^** ± 0.02	1.07**^b^** ± 0.07	1.32**^a^** ± 0.033	1.16**^b^** ± 0.03	1.22 ± 0.02	1.14 ± 0.03
28.-El_I_*	0.50 ± 0.01	0.44 ± 0.03	0.54 ± 0.008	0.48 ± 0.01	0.54**^a^** ± 0.01	0.45**^b^** ± 0.03
29.-Ew_I_*	0.68 ± 0.01	0.61 ± 0.04	0.71**^a^** ± 0.010	0.65**^b^** ± 0.01	0.73 ± 0.01	0.63 ± 0.04
30.-HL_I_*	0.98**^a^** ± 0.02	0.83**^b^** ± 0.06	0.98 ± 0.019	0.86 ± 0.01	0.95 ± 0.01	0.81 ± 0.06
31.-HW_I_*	1.47**^a^** ± 0.01	1.22**^b^** ± 0.08	1.48 ± 0.033	1.33 ± 0.02	1.45 ± 0.02	1.32 ± 0.02
32.-LLMP_I_*	0.72**^a^** ± 0.02	0.58**^b^** ± 0.04	0.76 ± 0.020	0.68 ± 0.02	0.73 ± 0.02	0.63 ± 0.04
33.-BL_I_*	8.39**^a^** ± 0.07	7.45**^b^** ± 0.51	8.56 ± 0.125	7.85 ± 0.16	8.66**^a^** ± 0.11	7.79**^b^** ± 0.54
34.-LAW_I_*	6.81**^a^** ± 0.04	6.04**^b^** ± 0.42	6.84 ± 0.075	6.31 ± 0.10	6.94 ± 0.06	6.35 ± 0.44
35.-**WLH_d_**	0.14 ± 0.00	0.15 ± 0.00	0.14 ± 0.002	0.15 ± 0.00	0.14 ± 0.00	0.14 ± 0.01
36.-**WLP_d_**	0.11 ± 0.00	0.11 ± 0.00	0.11 ± 0.001	0.11 ± 0.00	0.11 ± 0.00	0.10 ± 0.01
37.-**WLS_d_**	0.06 ± 0.00	0.06 ± 0.00	0.05 ± 0.003	0.05 ± 0.00	0.05 ± 0.00	0.05 ± 0.00
38.-**WLC_v_**	0.10 ± 0.00	0.10 ± 0.01	0.10 ± 0.002	0.11 ± 0.00	0.10 ± 0.00	0.11 ± 0.01
39.-**PR_v_**	0.09 ± 0.00	0.10 ± 0.01	0.09 ± 0.003	0.10 ± 0.00	0.11 ± 0.00	0.10 ± 0.01
40.-**SR_v_**	0.27**^a^** ± 0.01	0.23**^b^** ± 0.02	0.24 ± 0.007	0.24 ± 0.01	0.25 ± 0.01	0.25 ± 0.02
41.-RCR_v_*	0.06**^a^** ± 0.00	0.05**^b^** ± 0.00	0.06 ± 0.001	0.06 ± 0.00	0.06**^a^** ± 0.00	0.05**^b^** ± 0.01
42.-**RBW_l_**	0.08 ± 0.00	0.08 ± 0.01	0.08 ± 0.001	0.08 ± 0.00	0.08 ± 0.00	0.08 ± 0.01
43.-**RPC_l_**	0.04 ± 0.00	0.04 ± 0.00	0.04 ± 0.001	0.04 ± 0.00	0.04 ± 0.00	0.04 ± 0.00
44.-**RE_l_**	0.05 ± 0.00	0.04 ± 0.00	0.05 ± 0.001	0.05 ± 0.00	0.05 ± 0.00	0.05 ± 0.00
45.-**HRAL_l_**	0.05 ± 0.00	0.05 ± 0.00	0.04 ± 0.001	0.04 ± 0.00	0.04 ± 0.00	0.04 ± 0.00
46.-**BLW_l_**	0.08 ± 0.00	0.08 ± 0.00	0.08 ± 0.001	0.08 ± 0.00	0.08 ± 0.00	0.08 ± 0.01

**Table 6.** Results from two-way ANOVA to compare the variation of measurements of morphological characteristics of three Mexican *Aeneolamia* spp. Abbreviations: **HWE_d_** Head width with eyes **HL_d_** head length in dorsal view, **PW_d_** pronotum width in dorsal view **PL_d_** pronotum length in dorsal view **SW_d_** scutellum width in dorsal view **SL_d_** scutellum length in dorsal view **PcL_v_** postclypeus length in ventral view **PCW_v_** postclypeus width in ventral view **Al_v_** anteclypeus length in ventral view **AW_v_** anteclypeus width in ventral view **SL_v_** stylet length in ventral view **SW_v_** stylet width in ventral view **PcW_v_** posterior coxa width in ventral view **PcL_v_** posterior coxa length in ventral view **BLW_v_** body length without wings in ventral view **PCL_l_** postclypeus length in lateral view **PCW_l_** postclypeus width in lateral view **EL_l_** eye length in lateral view **Ew_l_** eye width in lateral view **HL_l_** head length in lateral view **HW_l_** head width in lateral view **LLMP_l_** length of lateral margin pronotum in lateral view **BL_l_** body length including wings in lateral view **LAW_l_** length of the anterior wing in lateral view **WLH_d_** width-length radio of head in dorsal view **WLP_d_** width-length ratio of pronotum in dorsal view **WLS_d_** width-lengh radio of scutellum in dorsal view **WLC_v_** width-length radio of clypeus **PR_v_** postclypeus ratio in ventral view (width/length) **SR_v_** stylet ratio in ventral view (width/length) **RCR_v_** coxa ratio (width/length) **RBW_l_** ratio between body length with wings and length without wings **RPC_l_** postclypeus radio in lateral view (width/length) **REl** eye ratio in lateral view (width/length) **HRAL_l_** head ratio in lateral view (width/length) **BLW_l_** ratio between the length of body with wings and forewing length in lateral view.

Acronym	Species		Sex		Interaction	
	*F*	*P*	*F*	*P*	*F*	*P*
11.-**HWE_d_**	1.744	0.181	18.75	4.09E-05	0.5288	0.5913
12.-**HL_d_**	1.69	0.191	24.43	3.89E-06	0.7839	0.4599
13.-**PW_d_**	1.261	0.289	16.7	9.95E-05	0.532	0.5894
14.-**PL_d_**	2.074	0.132	15.43	0.0001752	0.4199	0.6585
15.-**SW_d_**	2.127	0.126	12.87	0.0005607	0.9456	0.3925
16.-**SL_d_**	7.015	0.002	24.03	4.64E-06	3.41	0.03773
17.-**PcL_V_**	1.247	0.293	17.76	6.27E-05	0.3953	0.6747
18.-**PCW_v_**	1.284	0.282	37.3	3.03E-08	1.751	0.1799
19.-**Al_v_**	4.34	0.016	14.45	0.000272	0.8141	0.4465
20.-**AW_v_**	2.79	0.067	41.35	7.38E-09	0.07588	0.927
21.-**SL_v_**	3.333	0.041	14.03	0.0003309	0.9593	0.3874
22.-**SW_v_**	5.43	0.006	10.72	0.001547	0.362	0.6974
23.-**PcW_v_**	2.785	0.068	35.9	5.47E-08	0.5938	0.5546
24.-**PcL_v_**	0.8573	0.428	16.48	0.0001129	0.5701	0.5677
25.-**BLW_v_**	3.915	0.024	19.2	3.38E-05	0.923	0.4013
26.-**PCL_I_**	1.625	0.203	40.45	1.01E-08	1.033	0.3603
27.-**PCW_I_**	2.054	0.135	23.38	5.94E-06	1.596	0.2088
28.-**El_I_**	2.106	0.128	18.46	4.65E-05	0.4292	0.6524
29.-**Ew_I_**	1.037	0.359	12.17	0.0007772	0.2618	0.7703
30.-**HL_I_**	0.7906	0.457	22.52	8.46E-06	0.06886	0.9335
31.-**HW_I_**	1.068	0.348	30.43	3.75E-07	1.291	0.2803
32.-**LLMP_I_**	3.131	0.049	22.86	7.33E-06	0.6679	0.5155
33.-**BL_I_**	0.5747	0.565	10.47	0.001739	0.06293	0.939
34.-**LAW_I_**	0.3988	0.672	9.184	0.003245	0.1178	0.889
35.-**WLH_d_**	0.2057	0.815	0.1278	0.7216	0.4334	0.6498
36.-**WLP_d_**	0.2475	0.781	2.128	0.1484	0.543	0.583
37.-**WLS_d_**	1.834	0.166	1.272	0.2626	0.02747	0.9729
38.-**WLC_v_**	0.7389	0.481	2.875	0.09368	0.02094	0.9793
39.-**PR_v_**	2.144	0.124	2.638	0.1081	1.154	0.3202
40.-**SR_v_**	2.506	0.088	1.278	0.2615	4.683	0.01183
41.-**RCR_v_**	2.852	0.063	9.638	0.002617	0.9715	0.3828
42.-**RBW_l_**	1.17	0.315	0.551	0.46	0.3265	0.7223
43.-**RPC_l_**	0.4304	0.652	2.741	0.1015	0.1586	0.8536
44.-**RE_l_**	2.267	0.11	2.545	0.1144	1.71	0.1871
45.-**HRAL_l_**	0.2143	0.808	0.381	0.5388	0.4524	0.6377
46.-**BLW_l_**	0.8206	0.444	2.027	0.1582	0.6383	0.5307

### ﻿Multivariate analyses

To explore if the variation of morphological characteristics together segregates the specimens of *A.danpecki* sp. nov. in a discrete group within multidimensional spaces, a series of ordination analyses were performed. A principal coordinate analysis (PCoA) was performed from a Gower pairwise matrix among 28 male specimens using the ten discrete (male tegmina color pattern and male genitalia) and 36 continuous features. Also, three principal components analyses (PCAs) were performed to explore the geographical patterns of morphological variation among specimens using pairwise covariance matrices of 36 continuous characters. Additionally, we include canonical variate analyses (CVAs) to determine to what extent these features explained the possible taxonomic segregation based on the 90 specimens in males, females, and both sexes together. Multivariate analyses were performed considering each specimen as an operational taxonomic unit (OTU). Lastly, we looked for multivariate statistical differences among taxonomic groups of *Aeneolamia* recovered in the ordination analyses, with an analysis of similarities (ANOSIM) and the respective pairwise Hotelling’s T non-parametric tests among groups representing putative species. Groups recovered in the multivariate space were confirmed by the comparative morphological analysis of male genitalia.

### ﻿Geometric morphometry of aedeagus

From the male genitalia images that show the aedeagus intact, shape variation in patterns of aedeagus spines were quantified among *A.danpecki* sp. nov. (*n* = 4), *A.albofasciata* (*n* = 6), and *A.contigua* (*n* = 7) specimens using potential homologous landmarks (lm) and semi-landmarks (sml) (Bookstein 1991; Zelditch et al. 2004). The aedeagus shape was defined by two type I lm, and 16 sml. Semi-landmarks were defined using digital curves of equidistant points on photographs of aedeagus spines in lateral view with TPS tpsDig 1.40 software (Rohlf 2004). Semi-landmarks were specific sites located along the digital curvatures representing the outline of the aedeagus spine. Form configurations were digitalized as two-dimensional coordinates with tpsDig 1.40 software (Rohlf 2004). To remove scale effects, position, and orientation from configurations, and obtain shape coordinates, a generalized Procrustes analysis (Zelditch et al. 2004) was performed with the CoordGen6 program of IMP (Sheets 2003). The tangential variation of curvatures of shape coordinates was minimized using the minimum Procrustes distance criterion (Pérez et al. 2006). The highest proportion of shape variation in the data set was quantified by means of a relative warps analysis from adjusted coordinates (Zelditch et al. 2004). Shape variation was analyzed with the first three RWs and shape changes were visualized with Thin-Plate Spline technique by means of deformation grids.

### ﻿Geographical records

To illustrate the geographic distribution of *Aeneolamia* spp., the records of the analyzed specimens were projected onto a map of Mexican biogeographical provinces (Morrone et al. 2017).

## ﻿Results

In total, 46 morphological characters were evaluated: four discrete characters focused on tegmen color patterns in both sexes, six discrete characters on male genitalia, and 36 continuous characters were measured in specimens of both sexes: Six continuous quantitative morphological were reported by Rodríguez et al. (2002, 2003), and 30 new ones are proposed in this study.

### ﻿Discrete morphological characters

All tegmina and male genitalia features showed differences in character state frequencies among *A.danpecki* sp. nov., *A.albofasciata*, and *A.contigua* (Tables 2, 3). Two traits of the tegmen and four from the male genitalia exhibit exclusive character states for species: CAT, CDT, SGP, SEGP, SSP, and PRE.

*Aeneolamiadanpecki*: dark brown (22%) to black (78%) tegmen (Fig. 1A), the color of the internal clavus edge of the same color as tegmen (100%; “without color lines”) (Fig. 1D), the anterior third of the same color as tegmen (80%) (Fig. 1G) or with an inconspicuous, thin, transverse line (20%) (Fig. 1H), and the distal third of the same color (85%) (Fig. 1K) or with an incomplete transverse line (15%) (Fig. 1L) (Table 2); subgenital plates in ventral view: acute apex, acuminate in a pointed lobe and straight lateral edges (Fig. 2I); secondary subapical spine of parameres in lateral view: two rounded acute lobes similar in size and shape (Fig. 2L); aedeagus in lateral view: spines slightly sinuous, conspicuously curved upward and touching the superior margin of phallobase, tips bent to form an almost 90° angle (Figs 2O, 8A, B).

*Aeneolamiaalbofasciata*: dark brown (27%) to black (73%) tegmen (Fig. 1B), the color of internal clavus edge of the same color (85%) or with a yellowish or white line (15%) (Fig. 1E), the anterior third with a conspicuous broad transverse yellowish or white line (100%) (Fig. 1I), and the distal third with a complete broad transverse yellowish or white line (100%) (Fig. 1M) (Table 2); subgenital plates in ventral view: acute apex, acuminate in a rounded lobe and with straight lateral edges (Fig. 2J); secondary subapical spine of parameres lateral view: two acute lobes, the dorsal one conspicuously bigger than the ventral (Fig. 2M); aedeagus lateral view: spines slightly sinuous and tips slightly curved upward (Figs 2P, 8C).

*Aeneolamiacontigua*: light brown (30%) to dark brown (70%) tegmen (Fig. 1C), with an orange or red line in the internal clavus (100%) (Fig. 1F), the anterior third of tegmen with a conspicuous broad transversal orange or red line (100%) (Fig. 1J), and the distal third with a complete broad transversal orange or red line (100%) (Fig. 1N) (Table 2); subgenital plates in ventral view: obliquely truncate apex with a blunt distal edge and lateral edges slightly concave (Fig. 2K); secondary subapical of parameres in lateral view: with one prominent lobe (Fig. 2N); aedeagus in lateral view: spine sinuous and tips slightly curved downward (Figs 2Q, 8D).

#### Continuous quantitative morphological characters

Combining morphometric data of both sexes, *Aeneolamiadanpecki* is smaller than both *A.albofasciata* and *A.contigua* in most features analyzed except SW_d_, and HRAL_l_ (Table 4). Two-way ANOVA supported significant statistical differences among the species in six features: SL_d_ (*F*_SLd_ = 7.0; *p*_SLd_ ≤ 0.001), Al_v_ (*F*_Alv_ = 4.3; *p*_Alv_ ≤ 0.05), SL_v_ (*F*_Slv_ = 4.3; *p*_slv_ ≤ 0.05), SW_v_ (*F*_SWv_ = 5.4; *p*_SWv_ ≤ 0.05), BLW_v_ (*F*_BLWv_ = 3.9; *p*_BLWv_ ≤ 0.05), LLMPs (*F*_LLMPs_ = 3.1; *p*_LLMPs_ ≤ 0.05) (Tables 4, 6); multiple comparisons support that these measurements were lower in *A.danpecki* than in *A.albofasciata* or *A.contigua* (Table 4).

Two-way ANOVA also supported significant statistical differences between sexes in more than 20 features (Tables 5, 6). The interaction of “species” and “sex” factors was considered only to evaluate which features differed between sexes within each species (Tables 5, 6). In *A.danpecki*, 17 features were larger in females than males (HWE_d_, HL_d_, PW_d_, SW_d_, SL_d_, PL_d_, PcW_v_, AW_v_, SL_v_, PCW_v_, BLW_v_, PCL_I_, PW_s_, HL_s_, HW_s_, LLMP_s_, SRv); in *A.albofasciata* three (AW_v_, El_s_, PW_s_) were larger in females; and in *A.contigua* nine (HWE_d_, HL_d_, PcW_v_, Al_v_, AW_v_, PCW_v_, PcL_v_, PCL_I_, El_s_) were larger in females (Table 5).

### ﻿Multivariate analysis

The first two principal coordinates of PCoAs of continuous and discrete features of males explained 65% of variations (PCo1 = 39.99%, PCo2 = 15.01%) (Fig. 4A). Scatterplots of these principal coordinates (PCo1 vs. PCo2) showed that the specimens of *A.danpecki* and the other two *Aeneolamia* species fell into discrete phenotypic groups in the multivariate space analysis (Fig. 4A). The PCAs corresponding to the 36 characters combining both sexes (PCA_♂♀_), males alone (PCA_♂_), and females alone (PCA_♀_) explained more than 80% of the total variation in the first two principal components: PCA_♂♀_ = 99% (PC1_♂♀_ = 62.0%, PC2_♂♀_ = 37.0%); PCA_♀_ = 88.5% (PC1_♀_ = 72.5%, PC2_♀_ = 16.0%); PCA_♂_ = 98% (PC1_♂_ = 68.0%, PC2_♂_ = 30.0%). In the corresponding three-dimensional scatter plots, the specimens fell into three clusters in multivariate space corresponding to *A.albofasciata*, *A.contigua*, and *A.danpecki* (data not shown). The CVAs using 36 linear measurements explained more than 90% of the total variation in the first two canonical vectors in the three analyses performed; both sexes (CVA_♂♀_): CV1_♂♀_ = 64%, CV2_♂♀_ = 33%; males alone (CVA_♂_): CV1_♂_ = 81%, CV2_♂_ = 18%; and females alone (CVA_♀_): CV1_♀_ = 86% CV2_♀_ = 13%. The scatter plot between these variables showed that the specimens in well-differentiated phenotypic clusters correspond to *A.albofasciata*, *A.contigua*, and *A.danpecki* (Fig. 4B). Separate analyses by sex (CVA_♂_, CVA_♀_) displayed the clearest segregations of operational taxonomic units in well-defined discrete clusters (Fig. 4C, D); in the multivariate space, the OTUs corresponding to *A.danpecki* were the most distant and therefore more morphologically distinct from *A.albofasciata* and *A.contigua* than they were from one another. PERMANOVA supported statistically significant differences among groups displayed in CVA_♂♀_ (R_♂♀_ = 0.094; *p* ≤ 0.001), CVA_♂_ (R_♂_ = 0.094; *p* ≤ 0.001), CVA_♀_ (R_♀_ = 0.094; *p*_♀_ ≤ 0.001); pairwise comparisons supported differences among all constraints in the analyses of males and females alone: *A.danpecki* vs. *A.albofasciata* (*p*_♀_ ≤ 0.001, *p*_♂_ ≤ 0.001); *A.danpecki* vs. *A.contigua* (*p*_♀_ ≤ 0.05, *p*_♂_ ≤ 0.001), and *A.albofasciata* vs. *A.contigua* (*p*_♀_ = 0.1, *p*_♂_ ≤ 0.05); meanwhile, in the analysis of males and females together, multiple comparisons did not support differences between *A.albofasciata* vs. *A.contigua* (*p*_♂♀_ = 0.1). In the CVA_♂♀_ the discriminant function correctly classified 95.2% of OTUs according to the group to which they belong: one male of the new species was incorrectly classified as *A.contigua*, two males of *A.albofasciata* were classified as females of both *A.danpecki* and *A.contigua*, and one male of *A.contigua* was classified as *A.albofasciata*. Discriminant functions, analyzing the sexes separately, correctly classified 100% of the OTUs in both CVA_♂_ and CVA_♀_.

### ﻿Geometric morphometry of aedeagus

**Figure 5.**
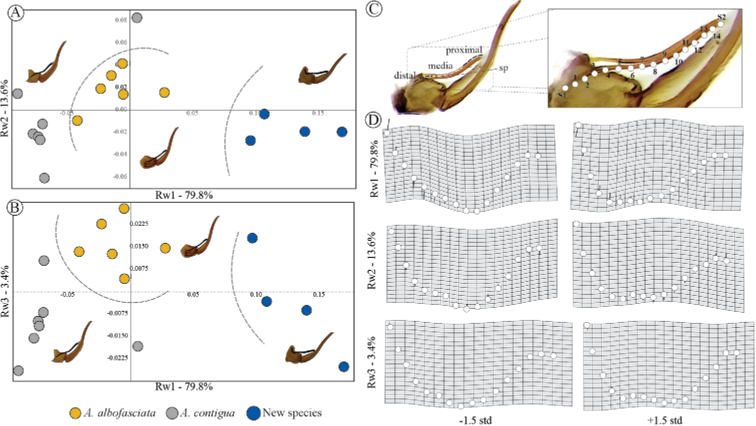
Scatter plots among the first three relative warps with its respective deformation grids ± 1.5 SD, corresponding to shape analysis of aedeagus spine (sp) of Mexican *Aeneolamia* spp. **A** Rw1 vs. Rw2 **B** Rw1 vs. Rw3 **C** position of landmarks (S1 and S2) and semi-landmarks (1–14) on aedeagus spine of *A.contigua***D** deformation grids ± 1.5 SD.

The superimposition of 15 aedeagus spine configurations of *Aeneolamia* members (*A.albofasciata*, *n* = 5; *A.contigua*, *n* = 6; *A.danpecki*, *n* = 4) showed that shape variation is found on both proximal and medial regions (Fig. 5). The first three Rws explained 96.8% of total variation (Rw1 = 79.8%; Rw2 = 13.6%; Rw3 = 3.4%). The respective two-dimensional scatterplot of these RWs displays three discrete groups corresponding to the three species (Fig. 5A, B). The deformations in the components Rw1, Rw2, and Rw3 were related to the curvature degree of proximal, medial, and distal areas of the spine, respectively (Fig. 5C, D). Specimens of *A.danpecki* showed a conspicuously curved proximal region upwardly bent to form an almost 90° angle, as was described in the character PRE.

Because our analyses support qualitative and quantitative discrete phenotypic variation among *Aeneolamia* species (two tegmina features characters and five genitalia ones) and the most pronounced morphological differences compared to the previously recognized species *A.albofasciata* and *A.contigua*, the specimens of the new species are grouped into a new taxon, *Aeneolamiadanpecki* Castro, Armendáriz, Utrera, sp. nov., described below.

#### ﻿ Aeneolamia danpecki

Taxon classificationAnimaliaHemipteraCercopidae

Castro, Armendáriz & Utrera sp. nov.

46B9DF3D-8B35-5596-9AB5-9C25650125C6

https://zoobank.org/7F8549F1-109F-4DA1-871C-0D95B2E3FD39

Figures 6 , 7

##### Type material.

**Figure 6.**
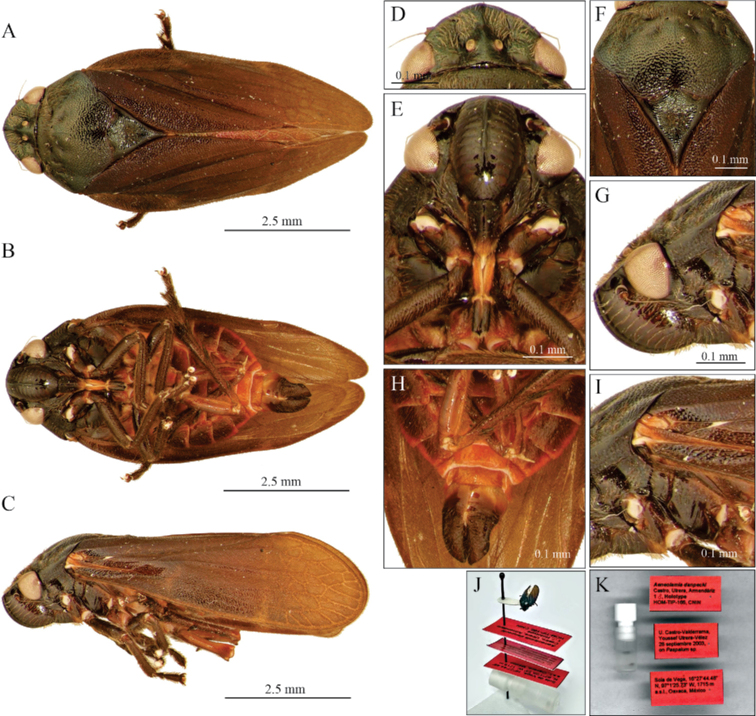
Male adult of *A.danpecki* (Holotype). Sola de Vega, Oaxaca **A** dorsal view **B** ventral view **C** lateral view **D** head in dorsal view **E** head in ventral view **F** prothorax in dorsal view **G** head in lateral view **H** abdomen in ventral view **I** anterior section of wing **J** mounted holotype **K** genital vial and labels of holotype.

***Holotype*.** HOM-TIP-166, 1 ♂ adult, coll. U. Castro-Valderrama and Youssef Utrera-Vélez leg., 28 September 2003, on *Paspalum* sp., Sola de Vega, 16°27'44.48"N, 97°1'25.73"W, 1715 m a.s.l., Oaxaca state, Mexico. Pinned adult deposited in CNIN (Fig. 6).

***Paratypes*.** HOM-TIP-167, 1 ♀, same data as holotype; 1 ♀, coll. Cervantes, A. Delgado, C. Mayorga, S. Gámez leg.; 5 km W San Martín Lachila, Mpio Zimatlán, Oaxaca, México, 16°35'39.18"N, 96°52'14.16"W, 12 July 2004. Pinned specimens deposited in CNIN. HOM-TIP-167, 32 ♀, 14 ♂ same data as holotype; 9 ♀, 6 ♂, coll. J. Romero Nápoles leg., 28 August 2018, on *Pennisetum* sp., La Trinidad Zaachila, Oaxaca, México, 16°55'03.84"N, 96°46'07.02"W, 1507 m a.s.l. Pinned specimens deposited in CEAM.

##### Etymology.

The epithet is a noun in the nominative singular standing in apposition to the genus *Aeneolamia*, in honor of Dr. Daniel C. Peck for his contributions to the knowledge of Cercopidae and his friendship with UC-V.

##### Diagnosis.

**Figure 7.**
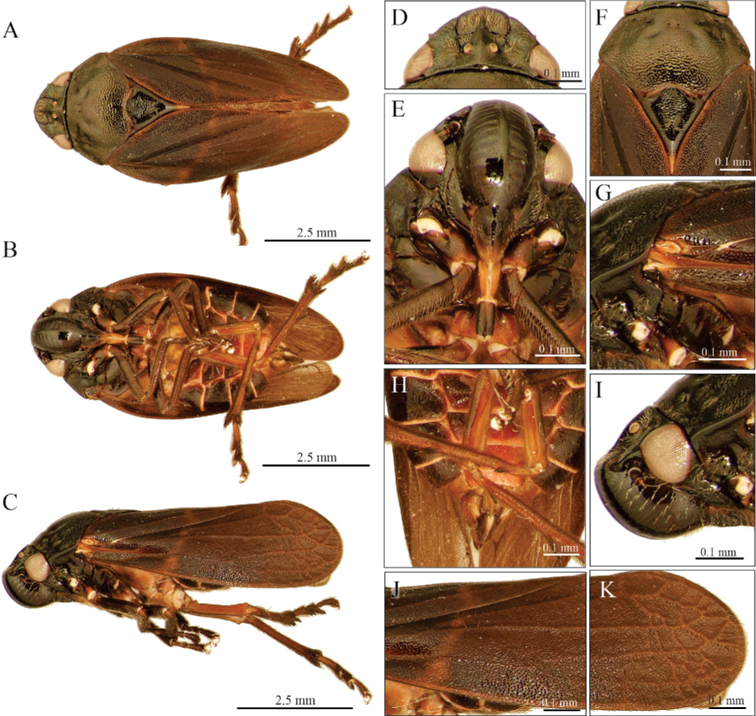
Female adult of *A.danpecki* (Paratype). Sola de Vega, Oaxaca **A** dorsal view **B** ventral view **C** lateral view **D** head in dorsal view **E** head in ventral view **F** prothorax in dorsal view **G** anterior section of wing **H** abdomen in ventral view **I** head in lateral view **J** median section of wing **K** distal section of wing.

**Figure 8.**
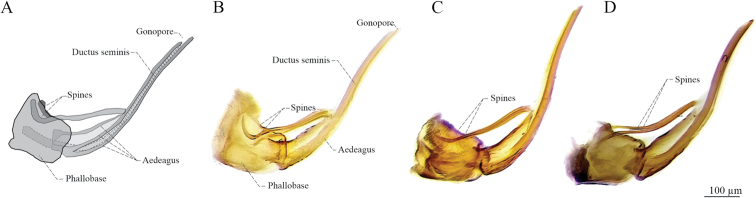
Lateral view of aedeagus of Mexicans *Aeneolamia* spp. **A, B***A.danpecki* (paratype) **C***A.albofasciata***D***A.contigua*.

*Aeneolamiadanpecki* Castro, Armendáriz, Utrera, sp. nov. is assigned to the genus *Aeneolamia* by virtue of its tubular aedeagus with a single pair of slender spines attached anteriorly near the middle of the shaft. It can be distinguished from the other known Mexican species of *Aeneolamia* by the following combination of characters: tegmen dark brown to black, with two incomplete and barely visible transverse bands, one oblique band on the basal third, and another straight band on the distal third or only basal band visible or both absent (Figs 6, 7); the apex of subgenital plates acute with an acuminate pointed lobe and straight lateral edges (Fig. 2I), the primary apical spine of parameres long and thin spine with a continuous curvature that is not angulated (Fig. 2F) and secondary subapical spine of parameres with two rounded acute lobes similar in size and shape (Fig. 2L); aedeagus spines slightly sinuous conspicuously curved upward and touching the superior margin of phallobase, tips bent to form an almost 90° angle (Figs 2O, 8A, B).

##### Description.

**Male measurements.** Lateral view length (*N* = 15) 7.45 ± 0.51 mm; width of head in dorsal view (*N* = 15) 1.75 ± 0.12 mm.

***Head*.** Dorsal view (Fig. 6A, D): black with brown setae; eyes black (discolored in figures); vertex black with median carina that originates in posterior margin of head and extends to tylus, a small depression between eye and median carina elongated and black, without setae, ocelli as close to each other as width of an ocellus; tylus quadrangular and black, with median carina. Ventral view (Fig. 6B, E): postclypeus black, inflated, with median carina black; anteclypeus black; basal segment of rostrum light brown in middle with black sides, distal segment black, reaching mesocoxae; antennae with scape and pedicel black to light brown, basal body of flagellum light brown, setae on pedicel scarce, flagellum brown, basal body of flagellum subcylindrical, smaller than pedicel and with arista. Lateral view (Fig. 6C, G): postclypeus black, convex, lateral grooves slightly marked.

***Thorax*.** Dorsal view (Fig. 6A, F): pronotum black with brown setae, punctate, hexagonal shape without carina, anterior zone with irregular depressed areas, one on each side, anterior margin straight, lateral anterior margin straight, lateral posterior margin slightly grooved, posterior margin grooved. Scutellum black, apex light brown in some specimens. Ventral view (Fig. 6B, E): with hind wing transparent light brown, venation brown-reddish, setae on both faces light brown; prosternum black to light brown, mesosternum black to light brown, metasternum light brown to reddish; fore legs dark brown, and meddle legs dark brown, with trochanters dark brown to light brown; hind legs with coxae, trochanters, femurs light brown with reddish tints or reddish, tibiae and tarsi dark brown to black; tibiae with two lateral spines and an apical crown with two rows of spines, basal spine small, distal spine 2 × longer than basal one, basal spine same size as apical crown spines; basitarsus with two rows of spines covered with scarce setae. Lateral view (Fig. 6C, I): pronotum not curved; tegmen dark brown to black, with two incomplete and barely visible transverse bands, one oblique band on basal third and another straight band on distal third or only basal band visible or both absent, the junction between Cu and R brown.

***Abdomen*.** Ventral view (Fig. 6B, H): black, except posterior and lateral edges of each sternite reddish, last sternite reddish and subgenital plates black or dark brown.

**Figure 9.**
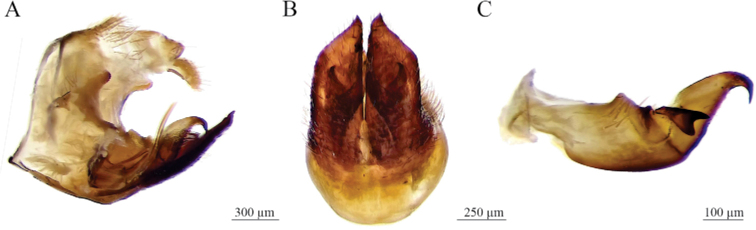
Genitalia of *A.danpecki* (paratype). Sola de Vega, Oaxaca **A** pygofer with phallobase, aedeagus and left paramere, lateral internal view **B** subgenital plates and parameres, ventral view **C** left paramere, lateral external view.

***Genitalia*.** Pygofer in lateral view (Figs 2C, 9A): lateral digital process, superior and inferior margins subequal in length, at the level of the inferior margin of the anal tube with the apex directed forward to the anal tube; subgenital plates in ventral view (Figs 2I, 9B) with lateral edge straight, interior margins parallel, not touching distally, wide along almost entire length, but not truncated apex, with shape acute, and tip acuminate with small hook. Paramere in lateral view (Fig. 9A, C): resting on subgenital plates, basal two-thirds broad and last third curved and tapered at tip to form a long hook, with two dorsal processes, one rounded mesal process with setae, another small process where the primary apical spine like-hook and the lateral secondary subapical spine converge, the primary apical spine long and slender with a continuous non-angulated curvature, sharp point and sclerotized; the lateral secondary subapical spine with two rounded lobes similar in size and shape, superior lobe sclerotized; inferior margin straight, distally curved to form a long spine like-hook. Aedeagus in anterodorsal view (Fig. 2R): bottle-shape with a thin apex, two thin, sinuous spines touching phallobase, tips as small hooks and hugging phallobase. Aedeagus in lateral view (Figs 2C, O, 8A, B, 9A): tubular, wide at base, abruptly narrowed where two lateral spines join shaft, lateral slightly sinuous spines touching superior margin of phallobase, and tips bent to form an almost 90° angle, apex acute, gonopore apical.

**Female measurements.** Lateral view length (*N* = 15) 8.39 ± 0.07 mm; width of head in dorsal view (*N* = 15) 2.06 ± 0.01 mm. Same characteristics as the male, except larger and posterior and lateral edges of each sternite light brown or reddish (Fig. 7A–K).

##### Distribution.

**Figure 10.**
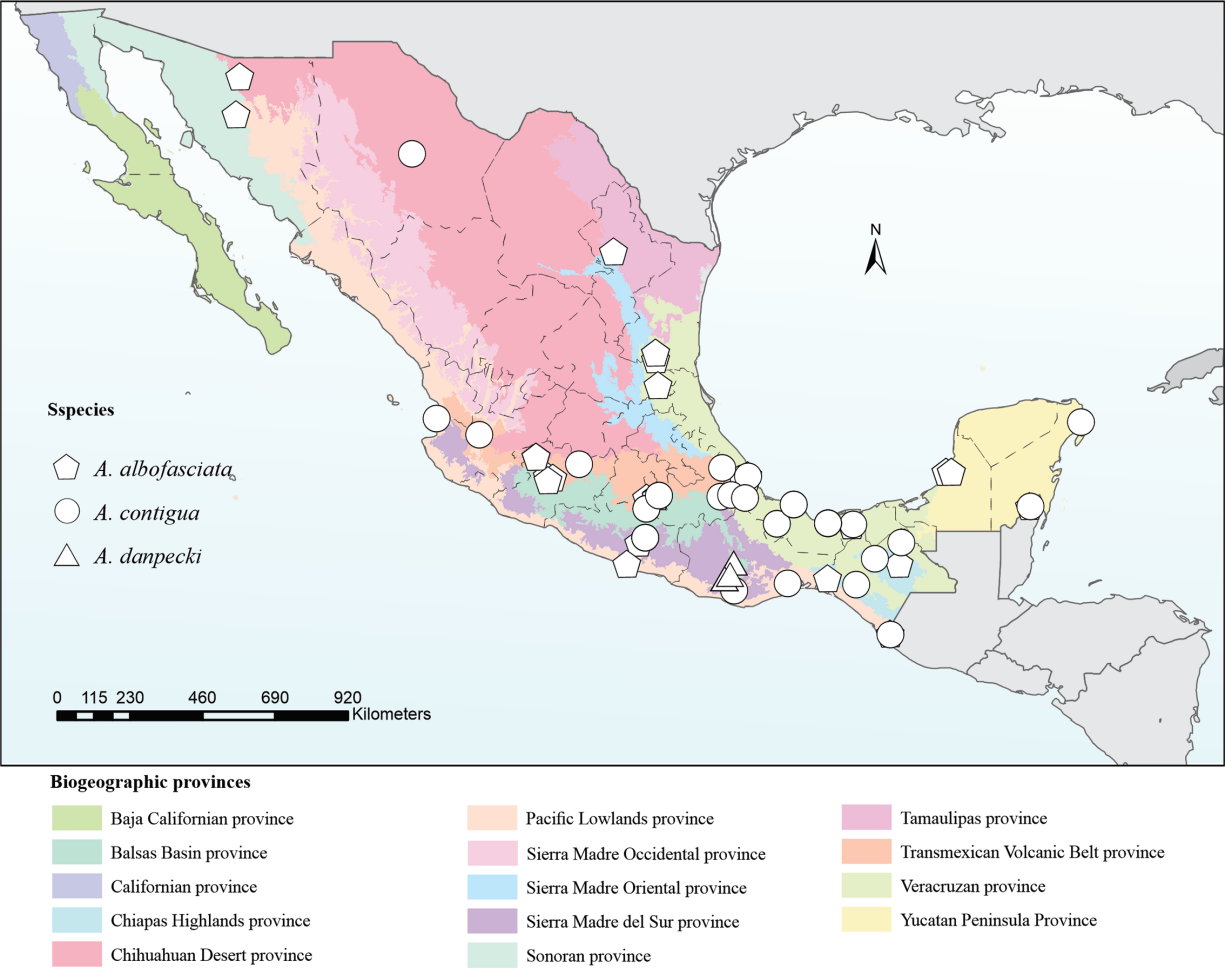
Geographical records of the three Mexican *Aeneolamia* species.

Oaxaca state, Mexico (Fig. 10).

##### Host.

*Paspalum* sp. and *Pennisetum* sp.

##### Remarks.

*Aeneolamiadanpecki* has black or dark brown subgenital plates with an acute end. In the type of material, San Martín Lachila is a municipality and not part of the Municipality of Zimatlán. *Aeneolamiadanpecki* was recognized as distinct for the first time as “Aeneolamiaaff.albofasciata (Lallemand, 1939)” by López-Posadas (2021: 63).

### ﻿Key to species and subspecies of *Aeneolamia* Fennah, 1949 from Mexico (based on Armendáriz-Toledano et al. 2022)

**Table:** 

1	Apex of subgenital plates obliquely truncate (Fig. 2K)	2
–	Apex of the subgenital plates acute (Fig. 2I, J)	3
2	Tegmen light brown to dark brown, with two orange transverse lines (Fig. 1J, N), with orange lines on claval edges V-shaped (Fig. 1F)	***A.contigua* (Walker, 1851)**
–	Tegmen black with narrow oblique transverse basal line interrupted at claval suture, a distal line straight, with lines on claval edges V-shaped (see Armendáriz-Toledano et al. 2022, fig. 13b); lines red in males, dark red in females	***A.contiguacampecheana* Fennah, 1951**
3	Tegmen dark brown to black, with one or two yellowish or white transverse lines (Fig. 1I, M), sometimes accompanied by lines on claval edges V-shaped (Fig. 1E), the secondary subapical spine of parameres with two acute lobes, the dorsal one conspicuously bigger than the ventral (Fig. 2M), aedeagus spines slightly sinuous and tips slightly curved upward (Figs 2P, 8C)	***A.albofasciata* (Lallemand, 1939)**
–	Tegmen dark brown to black, with two incomplete and barely visible transverse bands, one oblique band on basal third and another straight band on the distal third or only basal band visible or both absent (Figs 6, 7), the secondary subapical spine of parameres with two rounded acute lobes similar in size and shape (Figs 2L, 9C), aedeagus spines slightly sinuous, conspicuously curved upward and touching the superior margin of phallobase, tips bent to form an almost 90° angle (Figs 2O, 8A, B)	***A.danpecki* Castro, Armendáriz & Utrera, sp. nov.**

#### Geographic records

The distribution of *A.danpecki* was supported by three occurrence records from Sierra Madre del Sur province, in Oaxaca state (Fig. 10; Table 1). *Aeneolamiacontigua* had 34 occurrence records in Chihuahuan Desert (ChD), TVP, V, Pacific Lowlands (PL), and Yucatan Peninsula (YP) provinces in Chiapas, Chihuahua, Guerrero, Morelos, Nayarit, Oaxaca, Quintana Roo, Veracruz, and Yucatán states; and *A.albofasciata* was supported by 31 records in ChD, Sonoran, Sierra Madre Oriental, V, TVP, PL, and YP biogeographic provinces in Campeche, Chiapas, Guerrero, Michoacán, Morelos, Nuevo León, San Luis Potosí, Sonora, Tabasco, Veracruz, and Quintana Roo states. *Aeneolamiadanpecki* is sympatric with *A.contigua* and *A.albofasciata* in the Sierra Madre del Sur province. Meanwhile, *A.contigua* and *A.albofasciata* are distributed in almost all provinces except in the provinces of California and Baja California (Fig. 10).

## ﻿Discussion

### ﻿Discrete morphological characters

The evaluation of ten discrete characters of male tegmen and genitalia indicates that six of them (CAT, CDT, SGP, SEGP, SSP, and PRE) are useful to differentiate *A.danpecki*, and both sets of features together can differentiate this species from *A.albofasciata* and *A.contigua* as well as being diagnostic characters for *A.danpecki* (Tables 2, 3). The shape of subgenital plate apex, the shape of subapical spine of paramere, and the shape of the aedeagus spines of *A.danpecki* show unique character states (Figs 2I, L, O, 8A, B), and nothing similar was documented in the entire series of *A.albofasciata* and *A.contigua* examined. Additionally, the occurrence of diagnostic traits on the tegmen of both sexes allows reliable differentiation of both males and females of *A.danpecki* (Fig. 1A, D, G, H, K) from other species of the *Aeneolamia* (Fig. 1B, C, E, F, I, J, M, N). Regarding the tegmen, polymorphism is a common phenomenon in members of Cercopidae, with certain spittlebug species showing large variability in tegminal coloration patterns. Phenotypic variation in tegmen color among specimens within or among populations has been attributed to genetic causes (Aquino-Borges et al. 2020), resulting from differences in mating behavior, in attraction cues, or in geographic barriers (Hutchinson 1963; Farish and Scudder 1967). These factors have promoted highly diverse polymorphisms with dozens of morphotypes recognized throughout the species distribution in extreme cases (Farish 1972) and in others, only a few variants within and between localities (e.g., Paladini and Cavichioli 2015; Thompson and Carvalho 2016; Aquino-Borges et al. 2020). Members of the genus *Aeneolamia* are not exempt from this pattern, in which considerable color variation in tegmen has also been recognized in some species, within and among different spatially separated localities (Fennah 1949). For this reason, in a taxonomic sense, traits related to coloration patterns have been given less weight in defining taxa in this group than other body features (Paladini and Cavichioli 2013, 2015). As in other Cercopidae and *Aeneolamia* species, our analysis of discrete features in *A.danpecki*, *A.albofasciata*, and *A.contigua* allow us to recognize polymorphisms in the coloration patterns of the tegmen, reflected in that the species each presented traits with different character states (TC, CIE, CAT, CDT), and some of them were shared among the species (TC, CIE) (Table 2). However, the comparison of their frequencies supported the fact that the species have exclusive character states in two features, which constitute diagnostic features; in addition, they have different combinations of character states which together allow their recognition, at least in Mexico. The importance of the genital characters in Cercopidae studies was recognized by Fennah (1968) who stated that female and male genitalia characters can be used for grouping species. In comparison with the tegmen features of color, those characters of male genitalia have been shown to be conserved and therefore reliable for species identification and delimitation (Paladini and Cavichioli 2013, 2015; this study). In species with polymorphic tegminal color patterns, the specimens’ series display consistent discrete morphological features in different elements of male genitalia (Paladini and Cavichioli 2013; Paladini et al. 2018; Aquino-Borges et al. 2020). According to this pattern, our results show that elements of male genitalia easily discriminate males among the *Aeneolamia* analyzed (Figs 2, 8); despite the tegmen polymorphism found among them (Fig. 1), diagnostic characters of the genital plates, parameres, and aedeagus were found to be the same as in other *Aeneolamia* species (Paladini and Cavichioli 2013).

### ﻿Continuous quantitative morphological characters

The statistical analysis of morphological variation of Mexican members of *Aeneolamia* supports the earlier suggestion that specimens identified previously as A.aff.albofasciata in Armendáriz-Toledano et al. (2022) represent a new species, described here as *Aeneolamiadanpecki* Castro, Armendáriz, Utrera, sp. nov. Morphological differences in male genitalia (Fig. 2F–T) also support the species separation. *Aeneolamiadanpecki* exhibited smaller mean measurements than both *A.albofasciata* and *A.contigua* in the 36 features analyzed. Among these features Al_v_, BLW_v_, LAW_I_, PCW_v_, PW_d_, SW_d_, and SL_v_ displayed the most pronounced differences (Table 4).

### ﻿Multivariate analysis

From quantitative continuous and discrete characters (PCoA) of males and quantitative features of both sexes (PCA), permitted the recovery of discrete groups corresponding to the two previously recognized species, *A.albofasciata* and *A.contigua*, and the new species *A.danpecki* (Figs 4, 5), supporting that these cercopid species have strongly differentiated phenotypes. The robustness of the taxon clusters was demonstrated when the data set of characteristics was divided by sex, with *A.danpecki* being the most distant taxon in multivariate space and therefore morphologically distinct from *A.albofasciata* and *A.contigua*. In other hemipterans, quantitative measures and multivariate analysis have been used extensively to identify and delimit morphological variation within and between species (e.g., Blackman 1987; Gorla et al. 1993; Margaritopoulos et al. 2000; Jayasekera et al. 2010). This is the first time it has been utilized in Cercopidae. An outstanding result was that the multivariate analyses corresponding to each sex alone (CVA♂, CVA♀) displayed the clearest segregation of species (Fig. 4A, B). In the combined CVA of both sexes, one *A.contigua* female was grouped with *A.danpecki* and a male was grouped with *A.albofasciata* (Fig. 4B). This pattern can be explained by the sexual dimorphism of the three species studied. As in other cercopids, their females are usually equal to or somewhat larger than males of the same species (Paladini 2011), so most of the features measured in Mexican *Aeneolamia* females were larger than those of males. *Aeneolamiaalbofasciata* was the species with least pronounced sexual dimorphism in size, while *A.contigua* displays a greater difference between males and females (Fig. 4B). In other cercopids different sexually dimorphic traits have been recognized, such as ornamentation patterns in the tegmen (Peck et al. 2004; Paladini and Carvalho 2008), the profile of the anteclypeus (Hamilton 1977; Liang 2020), the form of anteclypeus in ventral view (Liang 2020), the tibial glands in male adults (Liang 2003, 2020), and an elongated basal body of the antenna in males of some genera of Ischnorhinini (Fennah 1968; Carvalho and Sakakibara 1989). Also, in some species, size dimorphism goes in the other direction, where males are smaller than females (Peck et al. 2002a, 2004; Rodríguez et al. 2002, 2003).

### ﻿Geometric morphometry of the aedeagus

Morphometric analyses have been poorly explored in Cercopidae and quantitative analyses of the shape of genital structures have not been performed previously in the family. However, in studies of other Cicadomorpha and other Cercopoidea, morphometric analyses have been used to recognize and delimit new species. In the genus *Cycloscytina* Martynov, 1926, shape analysis of the wing allowed to elucidate the species status of its members and support an extinct new species from the Triassic (Chen et al. 2022). In *Philaenus*, the species limits and distribution boundaries between *Philaenusspumarius* L., 1758 and *Philaenustesselatus* Stål, 1864, were established based on a classical morphometric analysis of aedeagus (Seabra et al. 2019). Our results of geometric morphometric analysis indicate that the shape of the genital structures is quantitatively different among Mexican species of *Aeneolamia*. The lack of overlap in the shape configurations of the aedeagus spine confirms that this anatomical structure is a robust diagnostic character useful in their identification (Fig. 5A–D).

### ﻿Geographical records

In this study, it is evident that, at the biogeographical level, *A.danpecki* sp. nov. is in sympatry with *A.contigua* and *A.albofasciata* in the Sierra Madre del Sur. However, the records of Mexican *Aeneolamia* species (Table 1) and some authors (López-Collado and Pérez-Aguilar 2012) do not support that *A.danpecki* coexists in the same localities with another congeneric species (Fig. 10). In addition, the distribution of *A.danpecki* is narrower than those of *A.contigua* or *A.albofasciata*, having been reported only within the eastern portion of the eastern Sierra Madre del Sur province, which corresponds to the central valleys and mountains between Sola de Vega and the city of Oaxaca, Oaxaca State (Fig. 10; Smith 1941; Arriaga et al. 1997; Santiago-Alvarado et al. 2016). This region is characterized by several endemic plants (Pinaceae, Bruceraceae, Cactaceae, Iridaceae, Poaceae), invertebrate animals (Amphypterigidae, Carabidae, Cordulidae, Curculionidae, Passalidae), and vertebrate taxa (Anguide, Cricetidae, Plethodontidae, Soricidae, Trochilidae) (Morrone et al. 2017). *Aeneolamiadanpecki* specimens were collected on *Paspalum* sp. and *Pennisetum* sp. grasses and, like many other spittlebug species, probably makes use of other native and introduced grasses in Oaxaca. Its pest status, if any, was not established in *Paspalum* sp. or *Pennisetum* sp. The new species *A.danpecki* represents the first new species taxon in *Aeneolamia* since the description of *A.albofasciata* by Lallemand in 1939.

## Supplementary Material

XML Treatment for Aeneolamia danpecki
